# Assay Development and Identification of the First *Plasmodium falciparum* 7,8-dihydro-6-hydroxymethylpterin-pyrophosphokinase Inhibitors

**DOI:** 10.3390/molecules27113515

**Published:** 2022-05-30

**Authors:** Marie Hoarau, Nattida Suwanakitti, Thaveechai Varatthan, Ratthiya Thiabma, Roonglawan Rattanajak, Netnapa Charoensetakul, Emily K. Redman, Tanatorn Khotavivattana, Tirayut Vilaivan, Yongyuth Yuthavong, Sumalee Kamchonwongpaisan

**Affiliations:** 1 National Center for Genetic Engineering and Biotechnology (BIOTEC), National Science and Technology Development Agency, Pathum Thani 12120, Thailand; nattida.suw@biotec.or.th (N.S.); thaveechai.var@biotec.or.th (T.V.); ratthiya.thi@biotec.or.th (R.T.); roonglawan@biotec.or.th (R.R.); netnapa.cha@biotec.or.th (N.C.); yongyuth@biotec.or.th (Y.Y.); sumaleek@biotec.or.th (S.K.); 2 Department of Chemistry, Faculty of Science, Chulalongkorn University, Bangkok 10330, Thailand; ekredman12@gmail.com (E.K.R.); tirayut.v@chula.ac.th (T.V.); 3 Center of Excellence in Natural Products Chemistry, Department of Chemistry, Faculty of Science, Chulalongkorn University, Bangkok 10330, Thailand; tanatorn.k@chula.ac.th

**Keywords:** malaria, HPPK, drug discovery, enzyme inhibitors, antifolates

## Abstract

In the fight towards eradication of malaria, identifying compounds active against new drug targets constitutes a key approach. *Plasmodium falciparum* 7,8-dihydro-6-hydroxymethylpterin-pyrophosphokinase (*Pf*HPPK) has been advanced as a promising target, as being part of the parasite essential folate biosynthesis pathway while having no orthologue in the human genome. However, no drug discovery efforts have been reported on this enzyme. In this study, we conducted a three-step screening of our in-house antifolate library against *Pf*HPPK using a newly designed *Pf*HPPK-GFP protein construct. Combining virtual screening, differential scanning fluorimetry and enzymatic assay, we identified 14 compounds active against *Pf*HPPK. Compounds’ binding modes were investigated by molecular docking, suggesting competitive binding with the HMDP substrate. Cytotoxicity and in vitro ADME properties of hit compounds were also assessed, showing good metabolic stability and low toxicity. The most active compounds displayed low micromolar IC_50_ against drug-resistant parasites. The reported hit compounds constitute a good starting point for inhibitor development against *Pf*HPPK, as an alternative approach to tackle the malaria parasite.

## 1. Introduction

Despite the global efforts deployed, malaria was still responsible for 627,000 deaths in 2020, most of them occurring in children under the age of 5. The fast appearance of resistance-inducing mutations has forced research to constantly renew its angle of attack, seeking novel drug targets, drug combinations and drugs with improved efficiency. Among the various available drug targets, multiple review articles reflecting on the future of antifolates against malaria have proposed *Plasmodium falciparum* 7,8-dihydro-6-hydroxymethylpterin-pyrophosphokinase (*Pf*HPPK) as promising [1,2,3,4,5].

**Figure 1:**
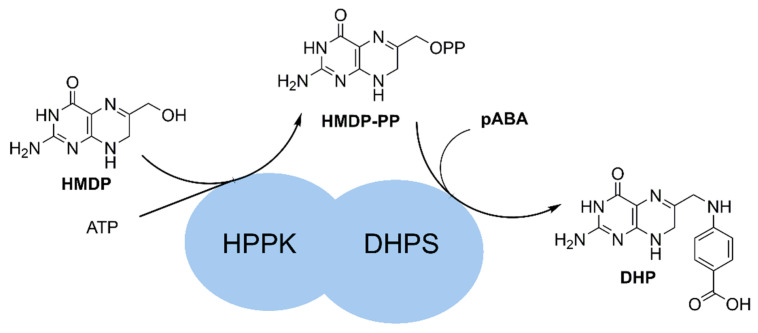
Reaction catalyzed by *Pf*HPPK-DHPS.

In protozoa, HPPK (EC 2.7.6.3) is located upstream dihydropteroate synthase (DHPS) and dihydrofolate reductase (DHFR) in the folate biosynthetic pathway. It is encoded by the *folK* gene as the N-terminal domain of the HPPK-DHPS bifunctional enzyme. HPPK is categorized as a pyrophosphate kinase and catalyzes the ATP-mediated diphosphate transfer to 2-amino-4-hydroxy-6-hydroxymethyl-7,8-dihydropteridine (HMDP) (Figure 1). In humans, kinase inhibitor design is made challenging by the large human kinome (>500 proteins), the highly conserved ATP binding pocket across the kinase family [6], and the high intracellular ATP concentrations. In comparison, *P. falciparum* has a relatively small kinome (<100 proteins), typically showing only 35–60% sequence identity to their mammalian orthologues, suggesting that selective inhibitor development is possible [7,8]. Successful inhibitor development has already been reported on the lipid kinase phosphatidylinositol-4-OH kinase (PI(4)K) [9].

Among kinases, *Pf*HPPK is of special interest because it has no orthologue in the human genome. Its HMDP substrate is not synthesized by human cells, while the pathway is essential for DNA synthesis in *P. falciparum*. This minimizes the selectivity issues and thus makes *Pf*HPPK an attractive drug target. For this reason, rather than targeting the ATP binding site, developing HMDP-competitive inhibitors of *Pf*HPPK appeared as a promising approach. An additional engaging feature of antifolates is their reported ability to work in synergy in the context of combination therapy. Exploiting a new folate pathway enzyme would open doors to new treatment opportunities. Despite these advantages, no drug discovery study has yet been reported on *Pf*HPPK.

To date, limited information is available on *Pf*HPPK. In a 2005 study, Jönsson et al. demonstrated that monofunctional *Pf*HPPK maintains its activity in a *E. coli* folK knockout complementation assay [10]. A subsequent study by Rattanachuen et al. confirmed that monofunctional *Pf*HPPK_1–350_ could be recombinantly expressed and purified, and it displays in vitro activity, although the activity is lower than in the bifunctional form [11]. A radioactive assay coupling *Pf*HPPK with *M. tuberculosis* DHPS demonstrated a K_m_ of 2.1 μM for HMDP substrate and 13.2 μM for ATP substrate for *Pf*HPPK, and a k_cat_ of 0.012 s^−1^. The study also demonstrated the importance of the nonhomologous region 74–86 for protein expression and enzymatic activity. Finally, the first crystal structure for *Pf*HPPK-DHPS was released in 2020 [12].

Because *Pf*HPPK and *Pf*DHPS catalyze successive reactions of the folate biosynthetic pathway, their active sites share similar substrate binding patterns. In order to identify inhibitors targeting specifically *Pf*HPPK, it was thus important to decouple the two protein domains.

Herein, we report the design of a new *Pf*HPPK-GFP fusion protein and its application for drug screening using GFP-tagged protein differential scanning fluorimetry (DSF-GTP). The DSF-GTP strategy, as developed by Schaeffer’s group [13], involves monitoring the denaturation of a protein of interest (POI)-GFP fusion by following GFP fluorescence. The denaturation of the POI affects GFP fluorescence and results in a denaturation peak that is sensitive to changes in the protein environment, including buffer composition and presence of ligands. This method was found useful for ligand screening in several case studies [14,15,16].

In this study, a combination of virtual screening, DSF-GTP and enzyme inhibition assay was used to screen our in-house antifolate library against *Pf*HPPK. The hit compounds identified constitute the first reported *Pf*HPPK inhibitors. Preliminary studies on hit compounds binding modes and in vitro ADME characterization of these hits were also completed.

## 2. Results

### 2.1. Design of the Protein Construct

For *E. coli* expression and purification of monofunctional *Pf*HPPK, the plasmid pET29a_PfHPPK361_GFP encoding for *Pf*HPPK gene as a fusion with GFPuv gene was constructed. *Plasmodium* proteins typically show low levels of expression in *E. coli*, requiring large volume of culture to obtain a few milligrams of purified protein. Fusion with GFP has been demonstrated to improve protein yields and stability for a number heterologous proteins, including *Pf*DHFR [17].

In the native *Pf*HPPK-DHPS protein, interdomain interactions were observed crystallographically between a DHPS loop (residues 519–523) and the HPPK helices α1 and αA [11]. Although *Pf*HPPK was found to be suitable for expression as a monofunctional enzyme, we hypothesize that the presence of a fusion protein at its C-terminus could improve its stability. We thus decided to fuse the GFPuv at the C-terminal end of *Pf*HPPK.

**Figure 2:**
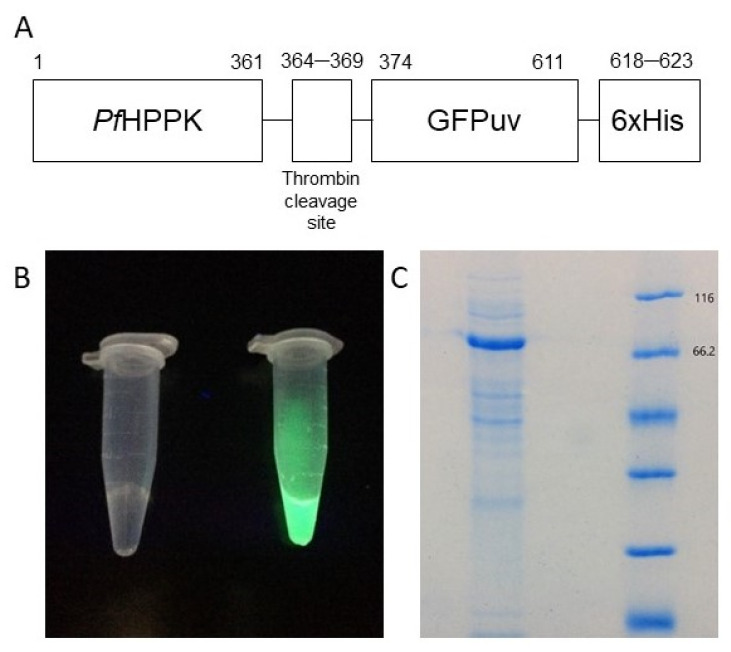
Cloning, expression and purification of *Pf*HPPK-GFP. (**A**) Schematic sequence of *Pf*HPPK-GFP. (**B**) UV-light exposed samples of buffer (**left**) and *Pf*HPPK-GFP (**right**). (**C**) SDS-PAGE of purified *Pf*HPPK-GFP.

*Pf*HPPK was fused to GFPuv through a 12-residue linker containing a thrombin cleavage site. Appended to the C-terminal end of the construct was a 6xHis tag to assist the protein purification (Figure 2A). The identity of the construct was confirmed by agarose gels with approximately 1 and 6.1 kb following digestion, and DNA sequencing. The corresponding protein was successfully expressed and purified from *E. coli* BL21 (DE3), with a yield ca. 1 mg protein per L of culture. Protein purity was estimated at 95% by SDS-PAGE analysis with a molecular weight of approximately 72.4 kDa (Figure 2C), and exposure of protein samples under UV light displays the green fluorescence characteristic of GFP (Figure 2B). The protein identity was also confirmed by LC-MS/MS (Figure S2).

### 2.2. Assay Development

In the development of the *Pf*HPPK-GFP construct, our goal was to have access to a method for cheap, fast, and specific ligand screening. The assay sensitivity should be sufficient to detect ligands with affinity up to high μM for identification of primary hit compounds.

The GFPuv is a laboratory-generated triple mutant of GFP with brighter fluorescence and an optimized codon usage for the expression in *E. coli*. Its fluorescence (λ_ex_ = 396 nm, λ_em_ = 507 nm) is suitable for detection using the FAM filter of a RT-PCR (λ_ex_ = 450–490 nm, λ_em_ = 510–530 nm) [15]. Compared to the standard SYPRO Orange DSF assay, the GTP-DSF assay is a more direct method, as it relies on the intrinsic fluorescence of the fusion protein. It can also perform on unpurified samples.

**Figure 3:**
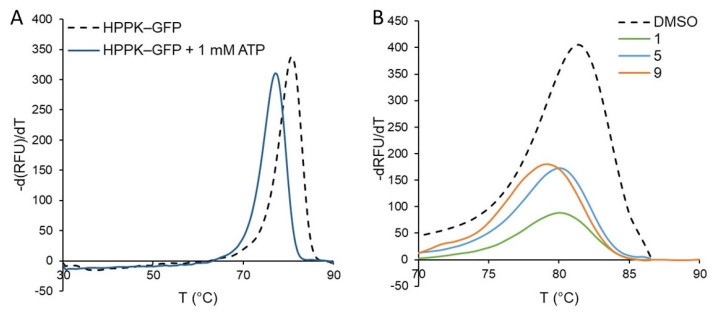
Typical melting temperature curves obtained by DSF-GTP for *Pf*HPPK-GFP in the presence of 1 mM ATP (**A**) and representative test compounds (**B**).

In a preliminary screening experiment, buffer conditions were investigated, searching for conditions that provide the largest thermal shift in the presence of 1 mM ATP. DSF curves were recorded under a total of 17 different conditions, with variations in buffer nature and pH, salts nature and concentration, effect of reducing agent, MgSO_4_ and glycerol. The optimal buffer was found to be phosphate buffer pH 7.2 50 mM, NaCl 50 mM, β-mercapto ethanol 1 mM, MgSO_4_ 5 mM, 20% glycerol, which corresponds to a T_m_ of 81 °C in the apo form and 80 °C in the presence of 1 mM ATP (Figure 3A).

The DSF curves obtained consistently showed a single denaturation peak, instead of two sequential peaks for *Pf*HPPK and GFP domains. A similar profile has been reported by Schaeffer’s group for at least five proteins from *E. coli*, *B. pseudomallei* and *S. aureus* [13], yet the unique peak shows the expected T_m_ variations in the presence of ATP (Figure 3A). In order to confirm that the change in T_m_ was not due to ATP binding to the GFP domain, a control experiment was conducted using GFP. Results show that although HPPK-GFP and GFP display similar DSF curves, no change was observed when adding up to 5 mM ATP to GFP (Figure S3). However, concentrations above 5 mM led to a small decrease in T_m_, likely due to nonspecific interactions. A concentration of 1 mM was thus selected for further screening assays.

### 2.3. Screening of the Antifolate Library

Having the ability to screen specifically for *Pf*HPPK binders, we were interested in identifying compounds to be used as starting points for drug development. Focusing on the HMDP binding pocket, we employed DSF-GTP to screen inhibitors against *Pf*HPPK from our in-house antifolate library. These compounds were prepared by our group over the years as drug candidates against *Pf*DHFR [18,19,20,21] and *Pf*DHPS (unpublished work) and were designed to bind to their pterin sites.

In a first step, virtual screening was completed on our 514-compounds antifolate library. For all compounds, molecular docking was conducted using Maestro software. The HMDP-bound *Pf*HPPK structure (PDB 6JWR) [12] was used as a receptor, and its protonation state was generated for a pH of 7.0 ± 0.1. Water molecules and bound ligands were removed, and the receptor structure was minimized. The binding site of interest was centered on co-crystallized AMP and encompassed the whole substrate pocket, and molecular docking was performed in standard precision mode. Docking poses were sorted by binding energy, and the 5% lowest scoring compounds were considered as hits, and binding poses were individually inspected (Table S1). Compounds were then clustered according to their core structure.

From there, protein binding properties of the hit compounds were assessed experimentally using the developed DSF-GTP assay. A total of 54 compounds were tested, including virtual screening hits and closely related structures. According to our protocol, each compound was mixed with *Pf*HPPK-GFP and submitted to a temperature gradient from 30 °C to 90 °C at 1 °C/min while GFP fluorescence was recorded. The first derivative of the resulting curve was used to determine the melting temperature (T_m_). For each set of experiments, a negative control containing DMSO was added, and ΔT_m_ values were calculated for each compound vs. the DMSO control. Among the compounds tested, 18 displayed a ΔTm values of −1 °C or lower. Two compound families were identified as the most promising by our DSF-GTP assay: 5-phenylazo-2,4-diaminopyrimidines (series 1) and phenyltriazolyl-2-amino-4-pyrimidinone (series 2). In series 2, linkers of different lengths that separate the phenyl triazolyl and the pyrimidine moieties were included.

For all the compounds displaying a measurable change of T_m_, *Pf*HPPK inhibition was recorded at 10 μM HMDP and ATP and 1 mM compound using the KinaseGlo assay. These conditions were chosen to be in the reported K_m_ range for both HMDP and ATP substrates [11,22], enabling detection of both competitive and uncompetitive inhibitors [23]. Compounds were incubated with HMDP, ATP and HPPK-GFP for 20 min at RT, followed by addition of the KinaseGlo reagent. After 10 min equilibration, luminescence was recorded, which directly reflects the quantity of ATP left in solution at the end of the reaction. Percentages of inhibition were calculated based on a DMSO positive control and a negative control lacking HPPK-GFP. Compounds structures, ΔT_m_ and levels of inhibition appear in Table 1 and Table 2.

**Figure 4:**
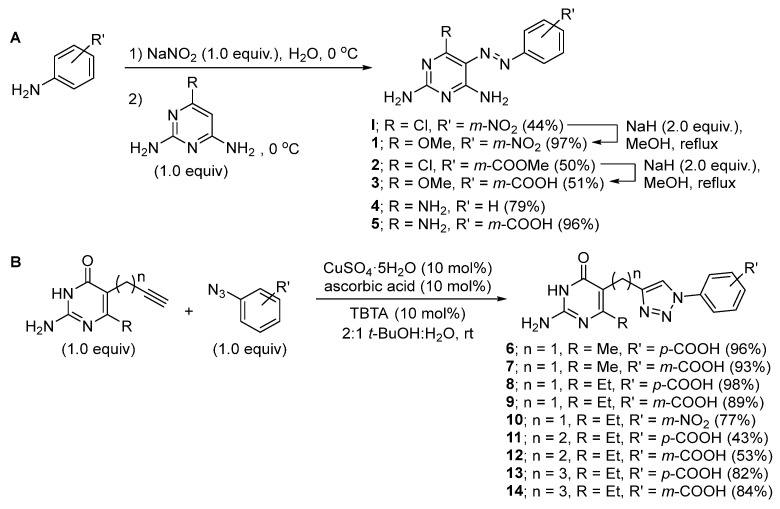
Synthesis of (**A**) 5-phenylazo-2,4-diamino pyrimidine derivatives (series 1); (**B**) phenyltriazolyl-2-amino-4-pyrimidinone analogs (series 2).

The 5-phenylazo-2,4-diamino pyrimidine derivatives were synthesized following the synthetic scheme illustrated in Figure 4A.

First, the diazonium coupling between substituted anilines and 2,6-diamino-4-chloropyrimidine or 2,4,6-triaminopyrimidine yielded the corresponding analogs (**I**, **2**, **4**, and **5**) in moderate to excellent yields. Next, nucleophilic aromatic substitution with MeOH mediated by NaH led to the replacement of Cl with OMe (**1** and **3**) in good yields. For the phenyltriazolyl-2-amino-4-pyrimidinone series, the click chemistry was performed between various aromatic azides and different pyrimidinones connected to an alkyne motif via linkers with 1–3 carbon lengths (Figure 4B). The corresponding analogs (**6–14**) were obtained in 43–98% yields.

In series 1, HPPK inhibition varies widely depending on R and R’ substituents. The best inhibition is obtained for the triamino pyrimidine **4** bearing no substituent on the phenyl ring, with 46% inhibition at 1 mM. In comparison, the m-COOH-substituted compound **5** shows poorer inhibition. For the 6-methoxy 2,4-diamino pyrimidine compounds **1** and **3**, a phenyl ring substitution with m-COOH (**3**) seems more favorable than m-NO_2_ (**1**). Finally, the 6-chloro 2,4-diamino pyrimidine compound **2** substituted with m-COOMe displays relatively poor inhibition.

In series 2, in general, changes in linker length and phenyl ring substitution affect HPPK inhibitory activity of the compounds. For compounds with one- (**6**, **7**, **8**, **9**, **10**) or three-carbon (**13** and **14**) linker between the pyrimidinone and triazole rings (R group), substitution with m-COOH is more favorable than with p-COOH. Changing of R” from methyl to ethyl further increases the enzyme inhibitory activity. Among these compounds, **14** with 3-carbon linker, m-COOH (R’) and Et (R”) is the most active, with 42.8% inhibition at 1 mM. Interestingly, in case of 2-carbon linker, p-COOH (**11**) is more favorable than m-COOH (**12**).

For the most potent compounds in each series, a dose-response inhibition experiment was conducted to determine IC_50_ values against the enzyme. Compound **14** showed IC_50_ of 814 ± 47 μM, while IC_50_ could not be determined for **4** due to limited solubility at high concentrations (Figure S5).

### 2.4. Molecular Docking

To gain insights into the mode of binding of the active compounds, all structures were studied by molecular docking using the high precision mode of Maestro software, in order to obtain higher resolution binding poses.

**Figure 5:**
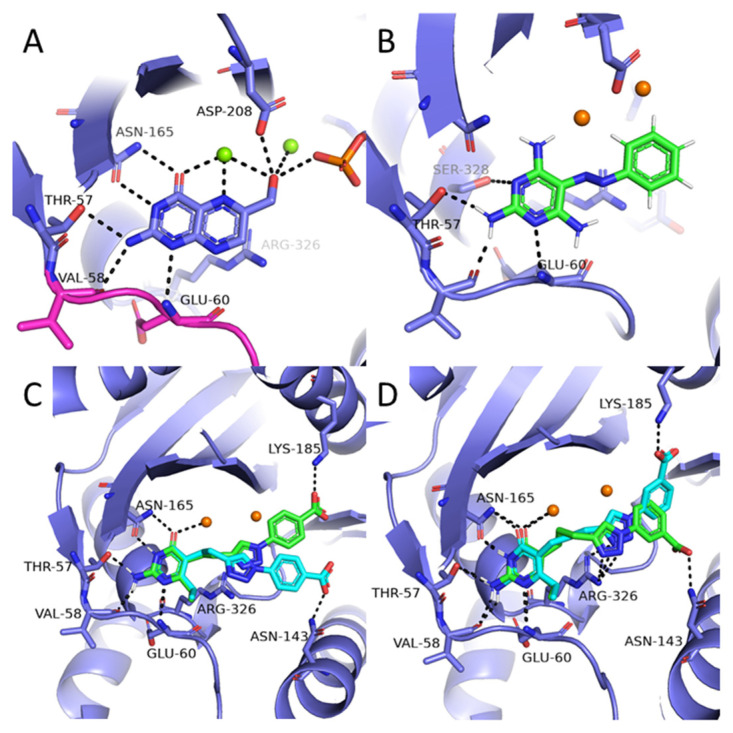
Binding mode of antifolates in HPPK active site. (**A**) Co-crystal structure of HMDP substrate in *Pf*HPPK (PDB 6JWR) [12]. Residues showing important conformational changes between apo and HMDP-bound form appear in pink. (**B**–**D**). Binding mode of compounds **4**, **11** and **14**, respectively, obtained by molecular docking. Two possible binding modes were proposed for compounds **11** and **14** and appear in green and cyan. Mg^2+^ ions were shown as spheres.

Compounds from series **(1)** bind to the *Pf*HPPK active site in a similar fashion as HMDP substrate, with interactions with Thr57, Val58, and Glu60 on the flexible loop (Figure 5).

However, the absence of a carbonyl group in this series prevents its interaction with Mg^2+^ and Asn165 (Figure 5B). In compounds **1** and **3**, such interaction is replaced by a polar contact between Asn165 and the 6-methoxy substituent (data not shown). In other compounds, the pyrimidine ring is tilted to form an H-bond interaction between a pyrimidine nitrogen N3 and Ser328. The nitrogen N7 of the diazo moiety occupies the same position as the pyrazine nitrogen of HMDP. The diazo group also forms π-π interaction with Arg326. Along the series, the positioning of the pyrimidine ring remains the same, but the presence of various substituents on the phenyl ring results in different degrees of twisting. In **4**, the unsubstituted phenyl ring is coplanar with the pyrimidine ring. In contrast, for **3** and **1**, the molecular docking results show that the phenyl ring forms a marked angle with the pyrimidine ring (66.6 and 86.7 degrees, respectively) to facilitate the interactions between the carboxylate/nitro groups and Arg326. In **2**, the phenyl ring forms an intermediate angle (17.7 degrees), and no polar contact involving the methyl ester group is observed. 

Compounds from the series (**2**) are anchored in the active site via three points. The 2-amino-4-pyrimidinone core forms a total of six polar contacts: with Mg^2+^ and Asn165 on one side, and with Thr57 Val58, and Glu60 on the flexible loop. This set of interactions is identical to the HMDP substrate. A second anchoring interaction is formed by the triazole ring and Arg326. This differs from the HMDP substrate binding mode, in which this residue does not form any polar interaction but interacts by π-π stacking with the pyrazine ring of HMDP. Finally, depending on the linker length and substituent position, the carboxylate group could form a H-bond interaction in the ATP binding pocket, either with Lys185 or with Asn143. These residues are not normally involved in the binding of the substrate. Overall, this series of compounds likely competes simultaneously with HMDP and ATP binding.

It is noteworthy that a similar molecular docking experiment was undertaken using the apo *Pf*HPPK crystal structure (PDB 6JWQ) [12] as a receptor. In this structure, loop 59–82 is in open conformation, leaving the active site open to bulk solvent (Figure S6). The molecular docking results obtained with this model were mostly inconsistent as the binding pocket was not sufficiently defined, and results were not considered.

### 2.5. In Vitro ADME, Cell Toxicity and Antiparasitic Activity 

It was important to profile the physicochemical and in vitro ADME properties of the hit compounds that were active against *Pf*HPPK to evaluate their suitability as lead compounds for further development. This allows us to identify the presence of metabolically unstable chemical groups and help us to select the most suitable candidate for further lead optimization. For representative compounds from the two series, cLog *S*, cLog *D*, cLog *P*, and polar surface area (PSA) values were calculated (Table 3). **11**, **13**, **14**, and **3** exhibit low lipophilicity, as attested by low cLog *D*_7.4_ and cLog *P* values, and high PSA. They are expected to show low cell permeability and gastrointestinal absorption. A similar trend was observed with the *Pf*DHFR inhibitor clinical candidate **P218** [24,25], due to the presence of the highly polar diaminopyrimidine and carboxylate. It is worthy to note that all compounds are negatively charged at pH 7.4, except for compound **4** which is neutral.

Intrinsic clearance was also measured in both human and rat liver microsomes in order to assess sensitivity to metabolic degradation. Except for compound **4**, all compounds show low intrinsic clearance in human and rat liver microsomal assay. This indicates that none of the chemical groups present in the compounds are sensitive to phase I metabolic enzymes. We hypothesize that the increased sensitivity of **4** to metabolic degradation is likely due to the presence of the unsubstituted phenyl ring, which is prone to metabolic modifications such as hydroxylation. All these compounds could also undergo phase II metabolism, such as carboxylate glucuronidation, that would not be detected in the present assay. However, experimental characterization of the metabolites would be needed to confirm this hypothesis.

In order to test compounds toxicity, cytotoxicity properties were measured against Vero cell line (African green monkey kidney cells) and KB cell line (human epithelial carcinoma cells). Following a standard protocol [19], cells were treated with variable concentrations of inhibitors for 72 h, and cell survival was measured by standard sulforhodamine B. In these conditions, no cytotoxicity was observed for any of the compounds, in either cell line (Table 4).

Finally, the various hit compounds were tested for their antimalarial activity against *P. falciparum* TM4/8.2 [26] and V1/S [27] strains. Parasite cultures were treated with test compounds at different concentrations, and antimalarial activity was measured using [3H]-hypoxanthine incorporation assay, according to our previously reported protocol [19]. As expected from the low level of enzyme inhibition, hits compounds showed little to no antimalarial activity (Table 4). Compound **4** displayed a IC_50_ of 75.4 μM against the drug-sensitive parasite, while compounds **1** and **2** displayed IC_50_ of 20.96 μM and 89.16 μM against the drug-resistant parasite, respectively. IC_50_ values could not be measured for other compounds. At this stage, there is no explanation for the difference in antiparasitic activity observed between TM4/8.2 and V1/S strains, and more investigations would be needed to conclude on this effect.

## 3. Discussion

From a medicinal chemistry perspective, *Pf*HPPK is an attractive drug target by the absence of a homologous enzyme in the human genome. Adding this to the low number of *Plasmodium* kinases, drug design based on *Pf*HPPK is expected to face few hurdles in terms of selectivity. Instead, the main challenge to overcome appears to be the enzyme-substrate binding affinity and specificity. In the crystal form, HMDP (14 non-H atoms) shows a total of 11 polar interactions, including 6 H-bonds with Asn165, Thr57, Val58, Glu60, and Asp208. An additional two coordination bonds with a Mg^2+^ ion, and three contacts with co-crystallized water molecules are observed. Finally, HMDP is further stabilized by π-π stacking interactions with Phe163 and Arg326 (Figure 5A). Thr57, Val58 and Glu60 are part of a flexible loop that alternates from an open conformation in the *apo* form to a closed conformation in the HMDP-bound form of the protein. Interaction between these residues and the substrate are likely essential for the enzyme activity. Altogether, this interaction pattern is finely tuned for HMDP and provides high specificity to the enzyme. To efficiently compete with the HMDP binding, we anticipate that inhibitors will have to display at least an equal number of interactions of similar strength. Compounds with a heteroaromatic ring with N-/O-rich substituents are thus likely to sustain the aforementioned interactions. Interaction with residues 57–60 is also desired to stabilize the protein in its closed form and prevent the substrate from accessing the active site. Considering these requirements, instead of using a diversity library, we chose to screen our in-house antifolate library. These compounds were designed to display competitive inhibition against downstream folate pathway enzymes dihydrofolate reductase (*Pf*DHFR) and/or dihydropteroate synthase (*Pf*DHPS) and contain a core structure mimicking the pterin ring of the folate precursors. Our 514-compound library was submitted to a three-step screening procedure, including virtual screening, DSF-GTP and enzymatic assay.

All hit compounds identified from our virtual screen contain either an amino pyrimidinone or a 2,4-diaminopyrimidine. Although the former would be expected, as being a direct substrate mimic, the latter was more surprising. Looking at the crystal structure, the double H-bond interaction between HMDP and Asn165 seems finely tuned for optimal interaction and would have been deemed essential, yet it appeared that these interactions can be replaced without significant loss of inhibition. In addition to this first anchoring group, our screen has highlighted the advantage of targeting Arg326, no longer through a π-π interaction but through H-bonds. A similar interaction pattern has been exploited in *Escherichia coli* HPPK [28] and DHPS [29] and *Bacillus anthracis* DHPS [30]. In our hits, interaction is achieved by a carefully positioned triazole ring using a suitable linker (Figure 5). Finally, according to molecular docking, the carboxylate groups could provide additional interactions with Asn143, Lys185, or Ser317. Due to the important variability across the series, it is difficult to conclude on the relative importance of these interactions. Of the three residues, only Ser317 is natively involved in substrate binding, forming two interactions with the adenine moiety of ATP. Developing molecules that target this residue could simultaneously prevent ATP and HMDP binding. Interaction with Asn143 would be an alternative strategy to occlude the entrance of the enzyme-active site, while stabilizing it in its closed conformation. 

The case of sulfa drugs has taught us that targeting loop regions may present risks, as loops generally show high susceptibility to resistance-inducing mutations [31,32]. Ideal new *Pf*HPPK drugs would restrict their interaction pattern to residues involved in substrate–protein interaction and limit additional loop interactions to backbone atoms. In this view, our initial hit compounds constitute good starting points for drug development. An additional matter to consider is the pharmacokinetics properties of the compounds. It is now widely recognized that early consideration of the pharmacokinetics helps us to avoid a number of pitfalls commonly encountered during drug design [33,34]. In particular, monitoring compounds’ metabolic stability allows us to identify undesired functional groups along the way. As the HMDP substrate presents unfavorable predicted physicochemical properties (clog *D*_7.4_ = −2.06, clog *P* = −3.6, clog *D* = −2.06, clog *S* = −1.0), the challenge resides in developing closely resembling compounds that display improved properties.

Our preliminary ADME profiling on five representative compounds shows low metabolic susceptibility against phase I metabolic enzymes for all but one compound. However, similar to the clinical drug candidate **P218**, the presence of carboxylate group is expected to cause rapid excretion through metabolic glucuronidation [25]. All hit compounds also display low lipophilicity and high PSA compared to standard recommendations. Future designs will focus on improving these properties, in addition to compounds affinity.

## 4. Experimental Methods

### 4.1. Synthesis

All reagents and solvents were obtained from Sigma-Aldrich (St. Louis, MO, USA), TCI chemicals (Tokyo, Japan), Fluorochem (Hadfield, Derbyshire, UK), and Merck (Darmstadt, Germany). All solvents for column chromatography from RCI Labscan (Samutsakorn, Thailand) were distilled before use. Reactions were monitored by thin-layer chromatography (TLC) using aluminum Merck TLC plates coated with silica gel 60 F254. Normal-phase column chromatography was performed using silica gel 60 (0.063–0.200 mm, 70–230 mesh ASTM, Merck, Darmstadt, Germany). Nuclear magnetic resonance spectra were recorded on a Bruker Avance (III) 400WB spectrometer (Bruker, Billerica, MA, USA) and JEOL JNM-ECZ500/S1 (500 MHz, JEOL, Tokyo, Japan). Chemical shifts were expressed in parts per million (ppm), and J values in Hertz (Hz). High-resolution mass spectra (HRMS) were obtained with a DART-TOF mass spectrometer (JEOL AccuTOF-DART) with electrospray ionization.

**(*E*)-6-Methoxy-5-((3-nitrophenyl)diazenyl)pyrimidine-2,4-diamine (1).** To a solution of 3-nitroaniline (276 mg, 2.0 mmol, 1.0 equiv.) in H_2_O (2.0 mL) and conc. HCl (0.8 mL) at 0 °C was added a solution of NaNO_2_ (138 mg, 2.0 mmol, 1.0 equiv.) in H_2_O (1.5 mL). The mixture was added to a solution of 2,6-diamino-4-chloropyrimidine (**I**, 298 mg, 2.0 mmol, 1.0 equiv.) in H_2_O (5.0 mL) at 0 °C, and then stirred at 0 °C for 1 h. The pH was adjusted to 6 using sat. NaHCO_3_ and the precipitate was collected by filtration, followed by washing with distilled water, cold EtOH and cold Et_2_O, and dried under vacuum to obtain *(E)*-6-chloro-5-((3-nitrophenyl)diazenyl)pyrimidine-2,4-diamine as an orange solid (300 mg, 44% yield). Next, **I** (147 mg, 0.5 mmol, 1.0 equiv.) was dissolved in MeOH (1.25 mL) and cooled to 0 °C. NaH (60% in mineral oil, 40 mg, 1.0 mmol, 2.0 equiv.) was slowly added, and the mixture was heated at reflux for 2 h. After completion, the mixture was quenched with H_2_O at 0 °C, and the precipitate was collected by filtration, followed by washing with distilled water, cold EtOH and cold Et_2_O, and dried under vacuum to obtain the title compound as a yellow solid (140 mg, 97% yield). ^1^H NMR (400 MHz, DMSO-*d_6_*) δ 9.49 (br s, 1H), 8.44 (s, 1H), 8.11 (t, *J* = 6.2 Hz, 1H), 7.92 (br s, 1H), 7.71 (t, *J* = 8.0 Hz, 1H), 7.19 (br s, 2H), 3.97 (s, 3H); ^13^C NMR (101 MHz, DMSO-*d_6_*) δ 168.6, 162.6, 156.0, 153.7, 148.7, 130.4, 127.7, 121.5, 114.3, 112.0, 53.8; HRMS (ESI+): *m*/*z* calcd for C_11_H_12_N_7_O_3_ [M+H]^+^ 290.1002, found 290.1518.

**Methyl *(E)*-3-((2,4-diamino-6-chloropyrimidin-5-yl)diazenyl) benzoate (2).** To a solution of 3-aminobenzoic acid (2.19 g, 16.0 mmol, 1.0 equiv.) in MeOH (80 mL) was added SOCl_2_ (4.76 g, 40.0 mmol, 2.5 equiv.) at 0 °C. The mixture was stirred at reflux for 8 h. After completion, the reaction was quenched using sat. NaHCO_3_, extracted with EtOAc, dried with anh. MgSO_4_, filtered and evaporated under vacuum. Purification with column chromatography (Hex/EtOAc = 1:4) yielded methyl-3-aminobenzoate as a brown oil (2.395 g, 99% yield). The title compound was synthesized with the same protocol as **I** using methyl-3-aminobenzoate (454 mg, 3.0 mmol, 1.0 equiv.), NaNO_2_ (207 mg, 3.0 mmol, 1.0 equiv.), and 2,6-diamino-4-chloropyrimidine (434 mg, 3.0 mmol, 1.0 equiv.) to give the title compound as an orange solid (456 mg, 50% yield). ^1^H NMR (400 MHz, DMSO-*d_6_*) δ 9.30 (br s, 1H), 8.37 (s, 1H), 8.18 (br s, 1H), 8.05 (d, *J* = 7.6 Hz, 1H), 7.97 (d, *J* = 7.6 Hz, 1H), 7.66 (t, *J* = 7.6 Hz, 1H), 7.52 (br s, 1H), 7.35 (br s, 1H), 3.90 (s, 3H); ^13^C NMR (101 MHz, DMSO-*d_6_*) δ 165.9, 164.8, 161.2, 155.9, 152.5, 130.8, 129.8, 129.4, 125.7, 122.0, 118.8, 52.3; HRMS (ESI+): m/z calcd for C_12_H_12_ClN_6_O_2_ [M+H]^+^ 307.0710, found 307.0687. 

***(E)*-3-((2,4-Diamino-6-methoxypyrimidin-5-yl)diazenyl) benzoic acid (3).** The title compound was prepared following the same protocol as **1** using **2** (215 mg, 0.7 mmol, 1.0 equiv.) and NaH (60% in mineral oil, 56 mg, 1.4 mmol, 2.0 equiv.) to give the title compound as a dark yellow solid (102.5 mg, 51% yield). ^1^H NMR (400 MHz, DMSO-*d_6_*) δ 9.49 (br s, 1H), 8.19 (s, 1H), 7.90 (d, *J* = 7.9 Hz, 1H), 7.87 (d, *J* = 7.9 Hz, 1H), 7.75 (br s, 1H), 7.58 (t, *J* = 7.9 Hz, 1H), 7.04 (br s, 2H), 3.97 (s, 3H); ^13^C NMR (101 MHz, DMSO-*d_6_*) δ 168.5, 167.2, 162.4, 156.0, 152.9, 131.8, 129.3, 128.3, 125.7, 120.8, 111.7, 53.6; HRMS (ESI+): m/z calcd for C_10_H_12_N_7_ [M+H]^+^ 230.1154, found 230.1232.

***(E)*-5-(Phenyldiazenyl)pyrimidine-2,4,6-triamine (4).** The title compound was prepared following the same protocol as **I**, using aniline (135 mg, 1.45 mmol, 1.0 equiv.), NaNO_2_ (100 mg, 1.45 mmol, 1.0 equiv.), and 2,4,6-triaminopyrimidine (182 mg, 1.45 mmol, 1.0 equiv.) to give the title compound as a yellow solid (262 mg, 79% yield). ^1^H NMR (400 MHz, DMSO-*d_6_*) δ 9.19 (br s, 2H), 8.45 (br s, 2H), 7.99–7.96 (m, 4H), 7.47 (t, *J* = 7.8 Hz, 2H), 7.37 (t, *J* = 7.7 Hz, 1H); ^13^C NMR (101 MHz, DMSO-*d_6_*) δ 153.3, 152.0, 129.0, 121.9, 106.4; HRMS (ESI+): m/z calcd for C_12_H_13_N_6_O_3_ [M+H]^+^ 289.1049, found 289.1183.

***(E)*-3-((2,4,6-Triaminopyrimidin-5-yl)diazenyl)benzoic acid (5).** The title compound was prepared following the same protocol as **I**, using 3-aminobenzoic acid (274 mg, 2.0 mmol, 1.0 equiv.), NaNO_2_ (138 mg, 2.0 mmol, 1.0 equiv.), and 2,4,6-triaminopyrimidine (250 mg, 2.0 mmol, 1.0 equiv.) to give the title compound as a yellow solid (525 mg, 96% yield). ^1^H NMR (400 MHz, DMSO-*d_6_*) δ 9.27 (br s, 2H), 8.53 (br s, 2H), 8.45 (s, 1H), 8.28 (d, *J* = 8.1 Hz, 1H), 8.05 (br s, 2H), 7.93 (d, *J* = 7.6 Hz, 1H), 7.58 (t, *J* = 7.4 Hz, 1H); ^13^C NMR (101 MHz, DMSO-*d_6_*) δ 167.0, 153.1, 152.1, 131.9, 129.5, 129.2, 124.9, 123.6, 106.7; HRMS (ESI+): m/z calcd for C_11_H_12_N_7_O_2_ [M+H]^+^ 274.1052, found 274.1068.

**2-Amino-6-methyl-5-((1-(4-(carboxy)phenyl)-1*H*-1,2,3-triazol-4-yl)methyl)pyrimidin-4(3*H*)-one (6).** To a mixture of CuSO_4_·5H_2_O (38 mg, 0.15 mmol) and ascorbic acid (26 mg, 0.15 mmol) in 5 mL of 2:1 *t*BuOH:H_2_O was added 2-amino-6-methyl-5-(2-propynyl)-4(3*H*)-pyrimidinone (34) (1.50 mmol) and 4-azidobenzoic acid (0.29 g, 1.80 mmol) in the presence of tris[(1-benzyl-1*H*-1,2,3-triazol-4-yl)methyl]amine (TBTA) (80 mg, 0.15 mmol). The reaction was stirred at room temperature for 24 h under nitrogen. After completion of the reaction, the solvent was removed under vacuum. Subsequently, the reaction was stirred at room temperature for 2 h following the addition of 3 mL of saturated aqueous solution of disodium salt of ethylenediamine tetra-acetic acid (Na_2_EDTA). The resulting solid was collected by filtration and rinsed with saturated Na_2_EDTA and water. The solid was dried under vacuum to give the desired product as an off-white solid (0.47 g, 96% yield). ^1^H NMR (500 MHz, DMSO-*d_6_*): δ 8.58 (s, 1H), 8.07 (d *J* = 8.5 Hz, 2H), 7.98 (d *J* = 8.5 Hz, 2H), 7.87 (br s, 2H), 3.75 (s, 2H), 2.23 (s, 3H). ^13^C NMR (126 MHz, DMSO-*d_6_*): δ 167.0, 161.6, 152.6, 147.1, 140.1, 131.6, 130.9, 121.0, 120.0, 110.8, 21.2, 18.0; HRMS (ESI+): m/z calcd for C_15_H_15_N_6_O_3_ [M+H]^+^ 327.1206, found 327.1224.

**2-Amino-6-methyl-5-((1-(3-(carboxy)phenyl)-1*H*-1,2,3-triazol-4-yl)methyl)pyrimidin-4(3*H*)-one (7).** 2-Amino-6-methyl-5-(prop-2-yn-1-yl)pyrimidin-4(3*H*)-one (1.50 mmol) was reacted with 3-azidobenzoic acid (1.80 mmol) following the same procedure as **6** to afford the desired product as an off-white solid (0.45 g, 93% yield). ^1^H NMR (500 MHz, DMSO-*d_6_*): δ 8.60 (s, 1H), 8.32 (s, 1H), 8.10–8.08 (m, 1H), 7.98–7.96 (m, 1H), 7.95 (br s, 2H), 7.67 (t, *J* = 7.9 Hz, 1H), 3.75 (s, 2H), 2.24 (s, 3H). ^13^C NMR (126 MHz, DMSO-*d_6_*): δ 166.9, 152.4, 146.8, 137.4, 133.0, 130.9, 129.5, 124.4, 121.1, 120.6, 111.0, 21.2, 17.8; HRMS (ESI+): m/z calcd for C_15_H_15_N_6_O_3_ [M+H]^+^ 327.1206, found 327.1197.

**2-Amino-6-ethyl-5-((1-(4-(carboxy)phenyl)-1*H*-1,2,3-triazol-4-yl)methyl)pyrimidin-4(3*H*)-one (8).** 2-Amino-6-ethyl-5-(prop-2-yn-1-yl)pyrimidin-4(3*H*)-one (1.50 mmol) was reacted with 4-azidobenzoic acid (1.80 mmol) following the same procedure as **6** to afford the desired product as an off-white solid (0.50 g, 98% yield). ^1^H NMR (500 MHz, DMSO-*d_6_*): δ 8.61 (s, 1H), 8.10 (br s, 2H), 8.07 (d, *J* = 8.6 Hz, 2H), 7.97 (d, *J* = 8.6 Hz, 2H), 3.77 (s, 2H), 2.57 (q, *J* = 7.5 Hz, 2H), 1.13 (t, *J* = 7.6 Hz, 3H). ^13^C NMR (126 MHz, DMSO-*d_6_*): δ 167.0, 161.3, 152.7, 147.1, 140.1, 131.6, 130.9, 121.2, 120.0, 110.4, 24.2, 20.9, 12.7; HRMS (ESI+): m/z calcd for C_16_H_17_N_6_O_3_ [M+H]^+^ 341.1362, found 341.1352.

**2-Amino-6-ethyl-5-((1-(3-(carboxy)phenyl)-1*H*-1,2,3-triazol-4-yl)methyl)pyrimidin-4(3*H*)-one (9).** 2-Amino-6-ethyl-5-(prop-2-yn-1-yl)pyrimidin-4(3*H*)-one (1.51 mmol) underwent click reaction with 3-azidobenzoic acid (1.80 mmol) following the same procedure as **6** and the desired product was obtained as an off-white solid (0.46 g, 89% yield). ^1^H NMR (500 MHz, DMSO-*d_6_*): δ 8.57 (s, 1H), 8.32 (s, 1H), 8.10–8.08 (m, 1H), 7.97–7.95 (m, 1H), 7.66 (t, *J* = 7.9 Hz, 1H), 7.50 (br s, 2H), 3.76 (s, 2H), 2.51 (q, *J* = 7.5 Hz, 2H), 1.09 (t, *J* = 7.5 Hz, 3H); ^13^C NMR (126 MHz, DMSO-*d_6_*): δ 166.9, 153.4, 147.6, 137.4, 133.0, 130.9, 129.5, 124.4, 121.1, 120.6, 110.0, 25.4, 21.1, 12.9; HRMS (ESI+): m/z calcd for C_16_H_17_N_6_O_3_ [M+H]^+^ 341.1362, found 341.2981.

**2-Amino-6-ethyl-5-((1-(3-(nitro)phenyl)-1*H*-1,2,3-triazol-4-yl)methyl)pyrimidin-4(3*H*)-one hydrochloride (10).** 2-Amino-6-ethyl-5-(prop-2-ynyl)pyrimidin-4(3*H*)-one (2.00 mmol) was reacted with 1-azido-3-nitrobenzene (2.4 mmol) according to the same procedure as **6** followed by subsequent treatment with 1 equiv. of concentrated HCl to afford the desired product as a yellow solid (0.58 g, 77% yield). ^1^H NMR (400 MHz, DMSO-*d_6_*) δ 8.78 (s, 1H), 8.66 (s, 1H), 8.38–8.35 (m, 1H), 8.31–8.29 (m, 1H), 8.26 (s, 2H), 7.88 (t, *J* = 8.2 Hz, 1H), 3.83 (s, 2H), 2.62 (q, *J* = 7.5 Hz, 2H), 1.19 (t, *J* = 7.5 Hz, 3H); ^13^C NMR (101 MHz, DMSO-*d_6_*) δ 160.6, 153.6, 152.1, 148.5, 146.6, 137.2, 131.5, 125.7, 122.9, 120.9, 114.3, 109.9, 23.6, 20.4, 12.1; HRMS (ESI+): m/z calcd for C_15_H_16_N_7_O_3_ [M+H]^+^ 342.1315, found 342.1316.

**2-Amino-6-ethyl-5-(2-(1-(4-(carboxy)phenyl)-1*H*-1,2,3-triazol-4-yl)ethyl)pyrimidin-4(3*H*)-one hydrochloride (11).** 2-Amino-6-ethyl-5-(but-3-ynyl)pyrimidin-4(3*H*)-one (1.50 mmol) was reacted with 4-azidobenzoic acid (1.80 mmol) according to the same procedure as **6** followed by subsequent treatment with 1 equiv. of concentrated HCl to afford the desired product as a brown solid (0.25 g, 43% yield). ^1^H NMR (400 MHz, DMSO-*d_6_*) δ ^1^H NMR (400 MHz, DMSO-*d_6_*) δ 12.73 (br s, 1H), 8.77 (s, 1H), 8.20 (s, 2H), 8.13 (d, *J* = 8.5, 1H), 8.02 (d, *J* = 7.5 Hz, 1H), 2.88–2.83 (m, 2H), 2.71–2.67 (m, 2H), 2.39 (q, *J* = 7.5 Hz), 1.05 (t, *J* = 7.5 Hz, 3H); ^13^C NMR (101 MHz, DMSO-*d_6_*) δ 166.4, 160.8, 152.3, 152.0, 147.2, 139.6, 131.1, 130.4, 120.9, 119.5, 111.7, 24.1, 24.0, 12.1; HRMS (ESI+): m/z calcd for C_17_H_19_N_6_O_3_ [M+H]^+^ 355.1519, found 355.1510.

**2-Amino-6-ethyl-5-(2-(1-(3-(carboxy)phenyl)-1*H*-1,2,3-triazol-4-yl)ethyl)pyrimidin-4(3*H*)-one hydrochloride (12).** 2-Amino-6-ethyl-5-(but-3-ynyl)pyrimidin-4(3*H*)-one (1.50 mmol) was reacted with 3-azidobenzoic acid (1.80 mmol) according to the same procedure as **6** followed by subsequent treatment with 1 equiv. of concentrated HCl to afford the desired product as a brown solid (0.31 g, 53% yield). ^1^H NMR (400 MHz, DMSO-*d_6_*) δ 12.68 (br s, 1H), 8.79 (s, 1H), 8.38 (s, 1H), 8.19 (s, 2H), 8.14–8.12 (m, 1H), 8.02 (d, *J* = 7.7 Hz, 1H), 7.73 (d, *J* = 8.0 Hz, 1H), 2.85 (t, *J* = 7.4 Hz, 2H), 2.69 (t, *J* = 7.5 Hz, 2H), 2.40 (q, *J* = 7.5 Hz, 2H), 1.05 (t, *J* = 7.5 Hz, 3H); ^13^C NMR (101 MHz, DMSO-*d_6_*) δ 166.1, 160.7, 152.2, 152.0, 147.1, 136.9, 132.6, 128.9, 123.9, 120.9, 120.1, 111.8, 24.1, 23.0, 12.1; HRMS (ESI+): m/z calcd for C_17_H_19_N_6_O_3_ [M+H]^+^ 355.1519, found 355.1510.

**2-Amino-6-ethyl-5-(3-(1-(4-(carboxy)phenyl)-1*H*-1,2,3-triazol-4-yl)propyl)pyrimidin-4(3*H*)-one hydrochloride (13).** 2-Amino-6-ethyl-5-(pent-4-ynyl)pyrimidin-4(3*H*)-one (1.50 mmol) was reacted with 4-azidobenzoic acid (1.80 mmol) according to the same procedure as **6** followed by subsequent treatment with 1 equiv. of concentrated HCl to afford the desired product as a brown solid (0.50 g, 82% yield). ^1^H NMR (400 MHz, DMSO-*d_6_*) δ 12.70 (br s, 1H), 8.71 (s, 1H), 8.15 (s, 2H), 8.13 (d, *J* = 8.6 Hz, 1H), 8.03 (d, *J* = 8.6 Hz, 1H), 7.73 (d, *J* = 8.0 Hz, 1H), 2.75 (t, *J* = 7.6 Hz, 2H), 2.53 (q, *J* = 7.6 Hz, 2H), 2.42 (t, *J* = 7.6 Hz, 2H), 1.84–1.76 (m, 2H), 1.18 (t, *J* = 7.5 Hz, 3H); ^13^C NMR (101 MHz, DMSO-*d_6_*) δ 166.4, 160.8, 151.9, 151.8, 148.2, 139.7, 131.0, 130.3, 120.3, 119.4, 112.7, 28.0, 24.6, 23.4, 23.0, 12.4; HRMS (ESI+): m/z calcd for C_18_H_21_N_6_O_3_ [M+H]^+^ 369.1675, found 369.1667.

**2-Amino-6-ethyl-5-(3-(1-(3-(carboxy)phenyl)-1*H*-1,2,3-triazol-4-yl)propyl)pyrimidin-4(3*H*)-one (14).** 2-Amino-6-ethyl-5-(pent-4-ynyl)pyrimidin-4(3*H*)-one (1.50 mmol) was reacted with 4-azidobenzoic acid (1.80 mmol) according to the same procedure as 6 followed by subsequent treatment with 1 equiv. of concentrated HCl to afford the desired product as a brown solid (0.51 g, 84% yield). ^1^H NMR (400 MHz, DMSO-*d_6_*) δ 12.70 (br s, 1H), 8.74 (s, 1H), 8.39 (s, 2H), 8.15–8.12 (m, 3H), 8.01 (d, *J* = 7.8 Hz, 1H), 7.73 (t, *J* = 7.7 Hz, 1H), 2.75 (t, *J* = 7.7 Hz, 2H), 2.53 (q, *J* = 7.6 Hz, 2H), 2.42 (t, *J* = 7.4 Hz, 2H), 1.83–1.76 (m, 2H), 1.18 (t, *J* = 7.6 Hz, 3H); ^13^C NMR (101 MHz, DMSO-*d_6_*) δ 166.3, 160.9, 152.0, 151.9, 148.0, 136.9, 132.5, 130.3, 128.9, 120.3, 120.1, 112.7, 28.0, 24.7, 23.4, 23.1, 12.4; HRMS (ESI+): m/z calcd for C_18_H_21_N_6_O_3_ [M+H]^+^ 369.1675, found 369.1666.

### 4.2. Cloning

The *Pf*HPPK and GFP sequences were PCR amplified from the existing pET29a-Pfhppk-dhps and pGFPuv [16], respectively, using the following primers:

5′-GCGGCATATGGAAACTATACAAGAACTAA-3′ (5′PfHPPK F),

5′-GCGGGTACCTTTCATCCTACTCA-3′ (3′PfHPPK 361 R), 

5′-GCGGATATCATGAGTAAAGGAGAAGAACTTTTC-3′ (5′GFP F),

5′-GCGGCG GCCGCTGATTTGTAGAG-3′ (3′GFP R).

The *Pf*HPPK amplicon from PCR reactions of 5′PfHPPK F/3′PfHPPK 361 R was digested with NdeI and KpnI. The digested PfHPPK fragment was cloned into pET29a linearized using the same enzymes, to obtain pET29a_PfHPPK361. Then, the GFP amplicon from the PCR reaction of 5′GFP F/3′GFP R was digested with EcoRV and NotI. Digested amplicon was cloned into pET29a_PfHPPK361 plasmids digested with the same enzymes. The resulting plasmid pET29a_PfHPPK361_GFP was used to transform *E. coli* DH5α cells, and plasmid sequence was verified by Sanger sequencing. Plasmid displaying expected sequence was then used to transform *E. coli* BL21(DE3) cells.

### 4.3. Protein Expression and Purification

*E. coli* BL21(DE3) cells carrying the pET29a_PfHPPK361_GFP plasmid were grown at 37 °C in LB media supplemented with kanamycin until the OD_600_ reached approximately 0.8. Protein expression was induced by addition of 0.4 mM IPTG and cells were cultured overnight at 20 °C. Cells were harvested and the cell pellet was stored at −20 °C. For purification, cells were thawed on ice in 25 mL lysis buffer (20 mM Tris buffer pH 8.0, 100 mM NaCl, 30 mM imidazole, β-mercapto ethanol 5 mM, 20% glycerol) and lysed by French Press. Lysate was then clarified by centrifugation and applied to a Ni^2+^-NTA column. Column was washed with lysis buffer, and protein was eluted using a gradient of elution buffer (20 mM Tris buffer pH 8.0, 100 mM NaCl, 300 mM imidazole, β-mercapto ethanol 5 mM, 20% glycerol). Fractions containing protein were concentrated using an Amicon 50 kDa filtration device. Concentrated protein was then loaded on a Q-Sepharose column equilibrated in buffer 1 (20 mM Tris buffer pH 8.0, 20 mM NaCl, 0.5 mM EDTA, β-mercaptoethanol 5 mM, 20% glycerol). Protein was eluted using a gradient of buffer 2 (20 mM Tris buffer pH 8.0, 500 mM NaCl, 0.5 mM EDTA, β-mercaptoethanol 5 mM, 20% glycerol). Fractions containing protein were concentrated using an Amicon 50 kDa filtration device and buffer exchanged with storage buffer (20 mM Tris buffer pH 8.0, 100 mM NaCl, 0.5 mM MgSO_4_, β-mercapto ethanol 5 mM, 20% glycerol). All steps were carried at 4 °C. Protein identity and purity were assessed by SDS-PAGE after each step, and protein concentration was assessed using Bradford assay. Protein aliquots were stored in −80 °C and thawed immediately before each experiment. Protein was also analyzed by proteomics at the National Omics Center (Thailand) to confirm protein sequence.

### 4.4. DSF-GTP

In a typical DSF-GTP experiment, a master mix containing buffer and *Pf*HPPK-GFP was prepared in a microcentrifuge tube, corresponding to a final protein concentration of 20 μg/mL. Ligands of interest were dispensed in low-profile 96-well plates (Bio-Rad) to 1 mM final concentration, and the master mix was dispensed to a final volume of 50 μL per well. In these conditions, DMSO content was maintained constant at 2%. The microplate was sealed with adhesive film and mixed by shaking for 2 min at 800 rpm at RT. The microplate was then submitted to a DSF run on a CFX96 RT-PCR (Bio-Rad, Hercules, CA, USA). DSF program was designed by starting with a 3 min equilibration phase at 30 °C, followed by a temperature gradient of 1 °C/min until 90 °C, recording fluorescence every 0.5 °C. Fluorescence was recorded using the FAM channel (λ_ex_ = 450–490 nm, λ_em_ = 510–530 nm). Curves were fitted using the Precision Melt Analysis software (Bio-Rad).

### 4.5. Enzymatic Assay

*Pf*HPPK-GFP inhibition assay was carried using the KinaseGlo Plus kit (Promega, Madison, WI, USA). In a black 96-well plate were dispensed test compounds (1 μL of 50 mM DMSO stock), master mix (100 mM Tris pH 9, 10 mM β-mercapto ethanol, 10 mM MgSO_4_, 0.01% *w*/*v* BSA, 10 μM HMDP and 10 μM ATP). Reaction was initiated by addition of 2 μg of enzyme, reaching a total reaction volume of 50 μL, and reaction was allowed to proceed for 20 min at RT shaking at 300 rpm. Reaction was then quenched by addition of 50 μL KinaseGlo reagent and allowed to equilibrate for 10 min at RT shaking at 300 rpm. Luminescence was recorded on a Biotek Synergy H1 plate reader (Agilent, Santa Clara, CA, USA) using an integration time of 1 s per well. Percentage of inhibition was calculated related to a positive control experiment (no enzyme added) and a negative control experiment (no inhibitor added).

### 4.6. Molecular Docking

Molecular docking experiments were conducted using Maestro program [35]. PDB file 6JWR was edited to remove water molecules and co-crystallized AMP ligand. Ligand structures were generated for pH 7.0 ± 1 and all tautomers were considered. Area of interest was centered on co-crystallized HMDP, which was omitted in calculations. Rigid molecular docking was performed using Glide [36]. For the screening step, the virtual screening default parameters were used, while for the docking step, high resolution default parameters were used. The top 5% scoring hits displaying the lowest binding energy were considered as hits and inspected manually. Structural graphics were drawn using PyMOL [37].

### 4.7. ADME Properties

Metabolic stability of test compounds was determined in human and rat liver microsomes. The assay was adapted from a previously published method [38]. Briefly, the test compounds were mixed with liver microsomes, and the metabolic reactions were initiated by the addition of NADPH. Acetonitrile containing an internal standard (labetalol) was added at 0, 5, 10, 30, and 60 min to stop the reactions. Samples were processed, and the remaining compounds in the reactions were detected using a LC-MS/MS. Distribution coefficient, solubility and polar surface area were predicted using Marvin software [39]. Partition coefficient was predicted using ACD/Labs software [40].

### 4.8. Cytotoxicity Testing

Compounds cytotoxicity properties were measured against Vero cell line (African green monkey kidney cells) and KB cell line (human epithelial carcinoma cells). Following a standard protocol [19]. In brief, KB cells (mouth epidermal carcinoma cells) were maintained in DMEM media supplemented with 10% fetal bovine serum, 3.7 g/L sodium bicarbonate and 1% MEM non-essential amino acids. Vero cells (African Green Monkey Kidney cells) were maintained in MEM/EBSS medium supplemented with 10% fetal bovine serum, 2.2 g/L sodium bicarbonate and 1 mM sodium pyruvate. For in vitro cytotoxicity assay, cells were treated with serial dilutions of the inhibitors for 72 h at 37 °C. Cell survival was measured using the sulforhodamine B assay and IC_50_ values were calculated.

### 4.9. Parasite Testing

Compounds were tested for their antimalarial activity against *P. falciparum* TM4/8.2 [26] and V1/S [27] strains according to our previously reported protocol [19]. In brief, parasite strains were maintained continuously in human erythrocytes in RPMI1640 supplemented with 25 mM HEPES pH 7.4, 0.2% NaHCO_3_, 40 µg/mL gentamicin and 8% human serum at 37 °C under 3% CO_2_. For in vitro antimalarial assay, parasite was treated with serial dilutions of the inhibitors, and parasite survival was determined using a modified microdilution radioisotope technique.

## 5. Conclusions

This work presents the first inhibitor screening against *Pf*HPPK. Using a *Pf*HPPK-GFP fusion protein specifically designed for screening purpose, we identified two series of compounds that readily bind *Pf*HPPK. The most potent compounds inhibit the enzyme in vitro in the μM range and are predicted to bind to the *Pf*HPPK active site in a similar fashion as the HMDP substrate. A preliminary ADME properties study shows good metabolic stability for six out of seven representative hit compounds in human and rat in vitro models. None of the fourteen hit compounds showed cytotoxicity in the two different mammalian cell lines tested, suggesting a favorable safety profile. At this stage, the most active compounds display μM-range activity against *P. falciparum* in vitro, and further drug development will be necessary. However, the present study constitutes a promising starting point for the elaboration of novel antifolates targeting *Pf*HPPK for the treatment of *P. falciparum* malaria.

## 6. Patents

The *Pf*HPPK-GFP protein construct was the object of a Thai petty patent (number KRRN 120042).

## Figures and Tables

**Table 1:** Structure and screening data for 5-phenylazo-2,4-diamino pyrimidine hit compounds (series 1).

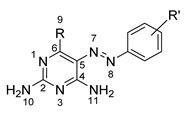				
Code	R	R’	ΔT_m_ (°C) ^1^	Inhib. at 1 mM (%) ^2^
**1**	OMe	*m*-NO_2_	−1 ± 0	2.5 ± 1.4
**2**	Cl	*m*-COOMe	−1 ± 0	11.0 ± 4.8
**3**	OMe	*m*-COOH	−1 ± 0	23.3 ± 4.8
**4**	NH_2_	H	−1 ± 0	46.0 ± 7.5
**5**	NH_2_	*m*-COOH	−1 ± 0	14.6 ± 3.2

^1^ Measured by DSF-GTP using *Pf*HPPK-GFP. ^2^ Measured by KinaseGlo assay kit using *Pf*HPPK-GFP.

**Table 2:** Structure and screening data for phenyltriazolyl-2-amino-4-pyrimidinone hit compounds (series 2).

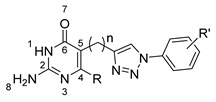					
Code	n	R	R’	ΔT_m_ (°C) ^1^	Inhib. at 1 mM (%) ^2^
**6**	1	Me	*p*-COOH	−1.0 ± 0	1.8 ± 0.6
**7**	1	Me	*m*-COOH	−1.0 ± 0	9.2 ± 1.5
**8**	1	Et	*p*-COOH	−1.2 ± 0.3	6.9 ± 2.0
**9**	1	Et	*m*-COOH	−1.0 ± 0	16.4 ± 2.9
**10**	1	Et	*m*-NO_2_	−1.2 ± 0.3	12.1 ± 4.0
**11**	2	Et	*p*-COOH	−1.3 ± 0.3	34.9 ± 5.5
**12**	2	Et	*m*-COOH	−1.3 ± 0.3	24.5 ± 2.3
**13**	3	Et	*p*-COOH	−1.5 ± 0	24.9 ± 3.1
**14**	3	Et	*m*-COOH	−1.2 ± 0.3	42.8 ± 5.5

^1^ Measured by DSF-GTP using *Pf*HPPK-GFP. ^2^ Measured by KinaseGlo assay kit using *Pf*HPPK-GFP.

**Table 3:** In silico and in vitro ADME properties of selected compounds.

Compound	cLog *S*_7.4_^1^	cLog *D*_7.4_^1^	cLog *P* ^2^	PSA ^1^	CL_int_ (HLM) (µL/min/mg)	CL_int_ (RLM) (µL/min/mg)
**P218**	−3.7	−0.20	2.79	133.58	<3	<3
**11**	−0.24	−1.23	2.11	135.49	<3	6.91
**13**	−0.50	−1.23	2.53	135.49	<3	7.38
**14**	0.00	−1.23	2.53	135.49	4.88	7.05
**3**	−1.50	−0.59	2.67	149.07	<3	<3
**4**	−2.95	1.83	0.83	128.56	362.63	82.39

^1^ Calculated with Marvin by ChemAxon. ^2^ Calculated with ACD/Labs. PSA: polar surface area, HLM: Human liver microsomes, RLM: Rat liver microsomes.

**Table 4:** IC_50_ values on *Plasmodium falciparum* strains TM4/8.2 (drug sensitive) and V1/S (drug resistant) and VERO and KB mammalian cell lines.

	IC_50_ (μM)			
Compound	TM4/8.2	V1/S	VERO	KB
**1**	>100	21.0	>100	>100
**2**	>100	89.2	>100	>100
**3**	>50	>50	>100	>100
**4**	75.4	>50	29.6	62.4
**5**	>10	>10	n.d.	n.d.
**6**	>25	>25	>25	>25
**7**	>25	>25	>25	>25
**8**	>50	>50	>50	>50
**9**	>50	>50	>50	>50
**10**	>50	>50	>50	>50
**11**	>50	>50	>50	>50
**12**	>50	>50	>50	>50
**13**	>50	>50	>50	>50
**14**	>50	>50	>50	>50

n.d.: not determined. When no IC_50_ could be determined, the highest tested concentration was noted.

## Data Availability

Not applicable.

## Supplementary Materials

The following supporting information can be downloaded at: https://www.mdpi.com/article/10.3390/molecules27113515/s1, Figure S1: Compounds characterization; Figure S2: Protein sequencing result for *Pf*HPPK-GFP; Figure S3: GFP melting temperature in the presence of various concentrations of A.T.P.; Figure S4: D.S.F. curves obtained for HPPK-GFP and GFP proteins; Table S1. Molecular docking scores obtained from antifolate library virtual screening; Figure S5: Dose-response inhibition for compound 14; Figure S6. Superimposition of *Pf*HPPK structures in the apo form and HMDP-bound form.

## Sample Availability

Samples of the DNA constructs are available from the authors upon request.
