# Supplementary material for: Assay Development and Identification of the First Plasmodium falciparum 7,8-dihydro-6-hydroxymethylpterin-pyrophosphokinase Inhibitors
Source: Molecules. 2022 May 30;27(11):3515. doi: 10.3390/molecules27113515 (PMC9182141; doi:10.3390/molecules27113515)

Supplementary materials

Figure S1. Compounds characterization

Signals at 3.4 ppm and 2.5 ppm on the ^{1}H NMR spectra correspond to water and DMSO, respectively. Signal at 40 ppm on the ^{13}C NMR spectra correspond to DMSO.

- ^{1}H (400 MHz, DMSO-d_6) and ^{13}C (101 MHz, DMSO-d_6) NMR spectra and DART-TOF HRMS spectrum of (*E*)-6-Methoxy-5-((3-nitrophenyl)diazenyl)pyrimidine-2,4-diamine (**1**)

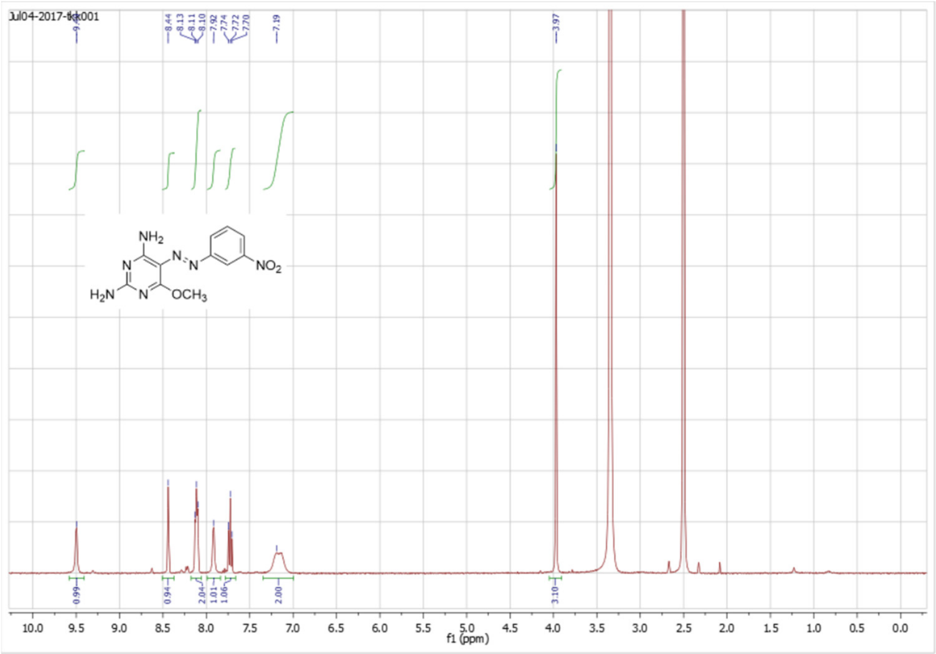

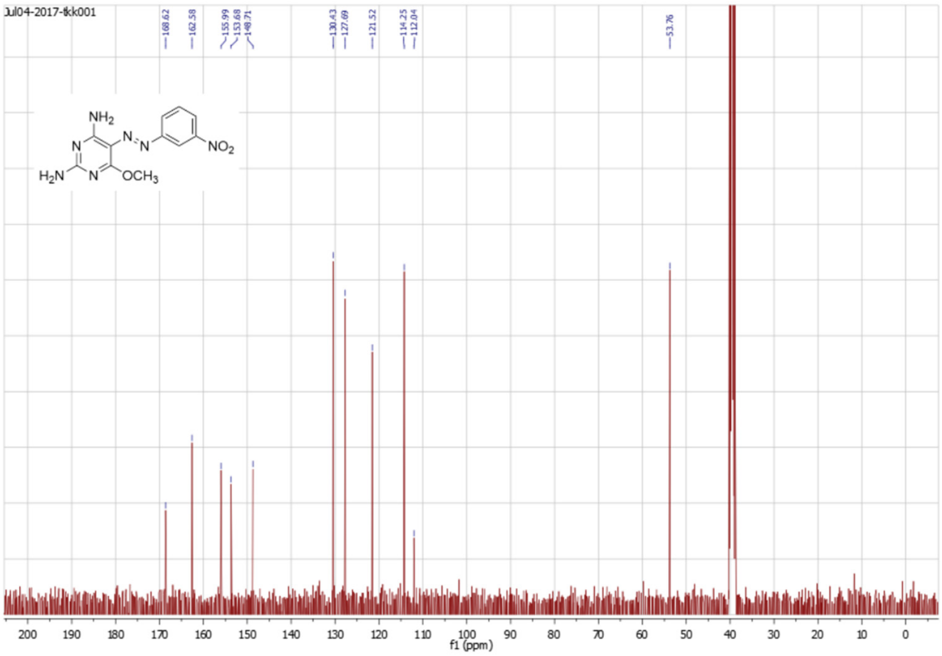
Jul04-2017-tkk001
168.62
162.58
155.99
153.68
148.71
130.43
127.69
121.52
114.25
112.04
53.76
NH2
N
N=N
NO2
H2N
N
OCH3
f1 (ppm)

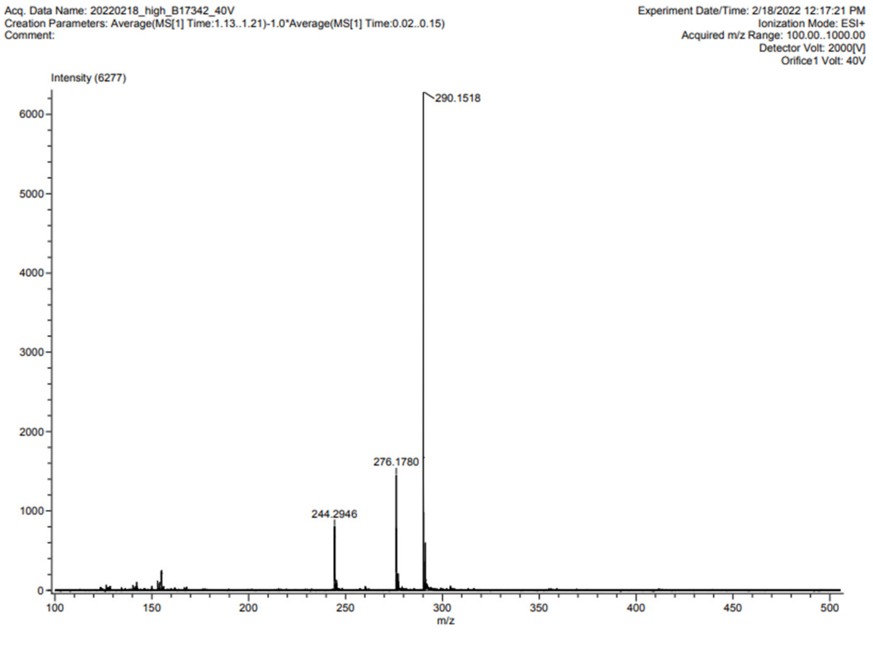
Acq. Data Name: 20220218_high_B17342_40V
Creation Parameters: Average(MS[1] Time:1.13..1.21)-1.0*Average(MS[1] Time:0.02..0.15)
Comment:
Experiment Date/Time: 2/18/2022 12:17:21 PM
Ionization Mode: ESI+
Acquired m/z Range: 100.00..1000.00
Detector Volt: 2000[V]
Orifice1 Volt: 40V
Intensity (6277)
290.1518
276.1780
244.2946
m/z

- ^{1}H (400 MHz, DMSO-d_6) and ^{13}C (101 MHz, DMSO-d_6) NMR spectra and DART-TOF HRMS spectrum of methyl (*E*)-3-((2,4-diamino-6-chloropyrimidin-5-yl)diazenyl)benzoate (**2**)

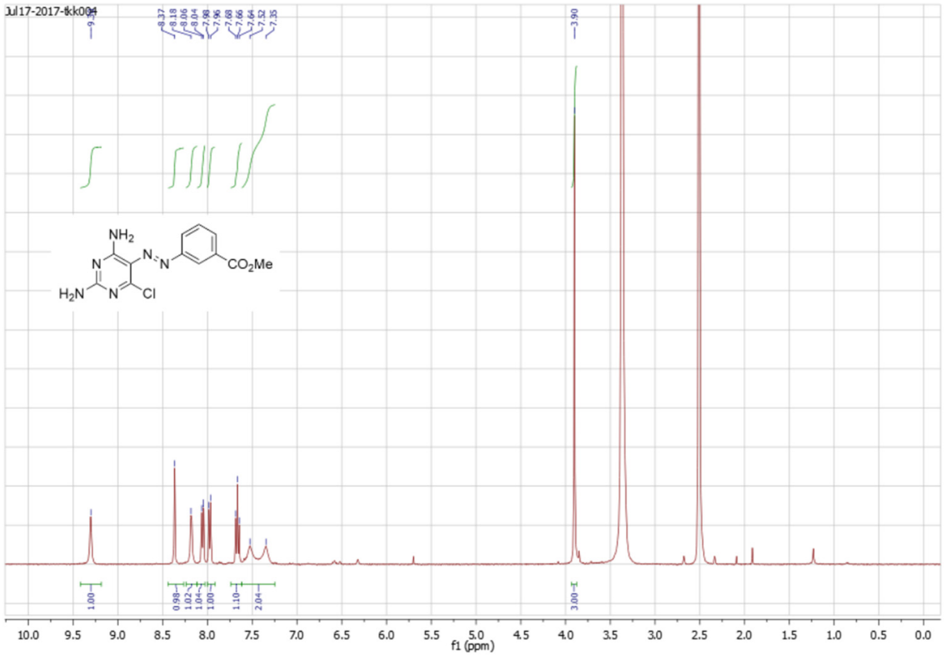

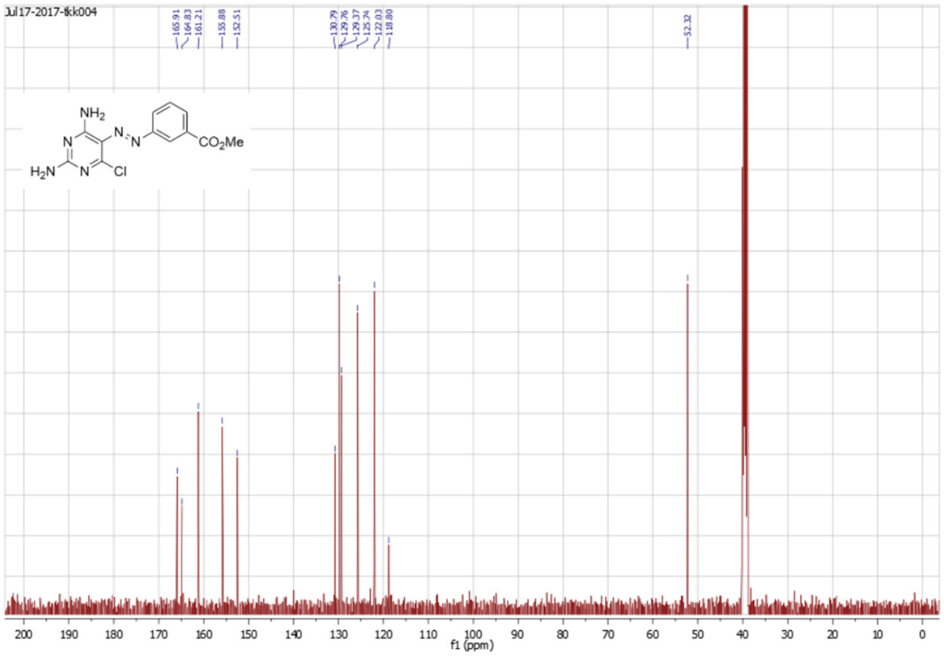

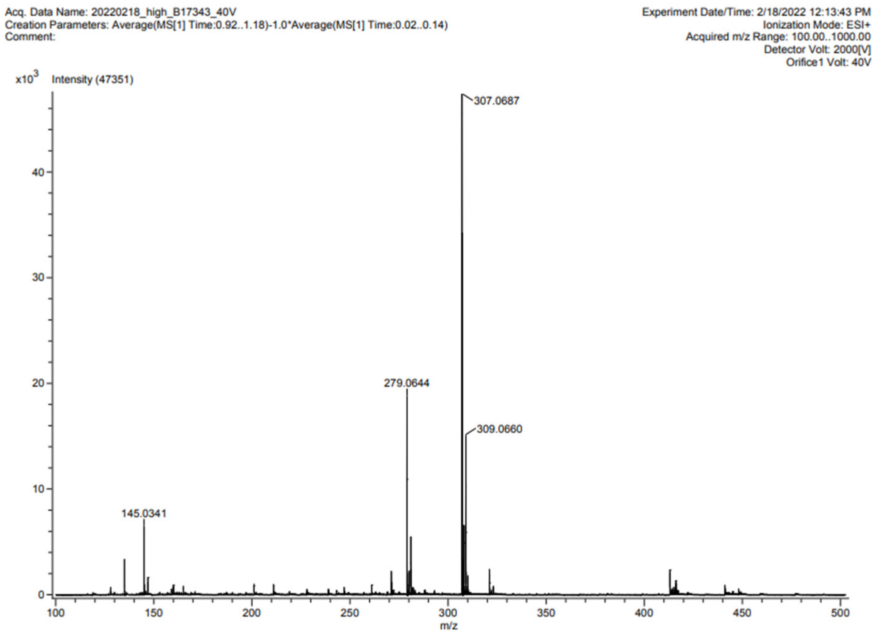

- ^{1}H (400 MHz, DMSO-d_6) and ^{13}C (101 MHz, DMSO-d_6) NMR spectra and DART-TOF HRMS spectrum of (*E*)-3-((2,4-diamino-6-methoxypyrimidin-5-yl)diazenyl)benzoic acid (**3**)

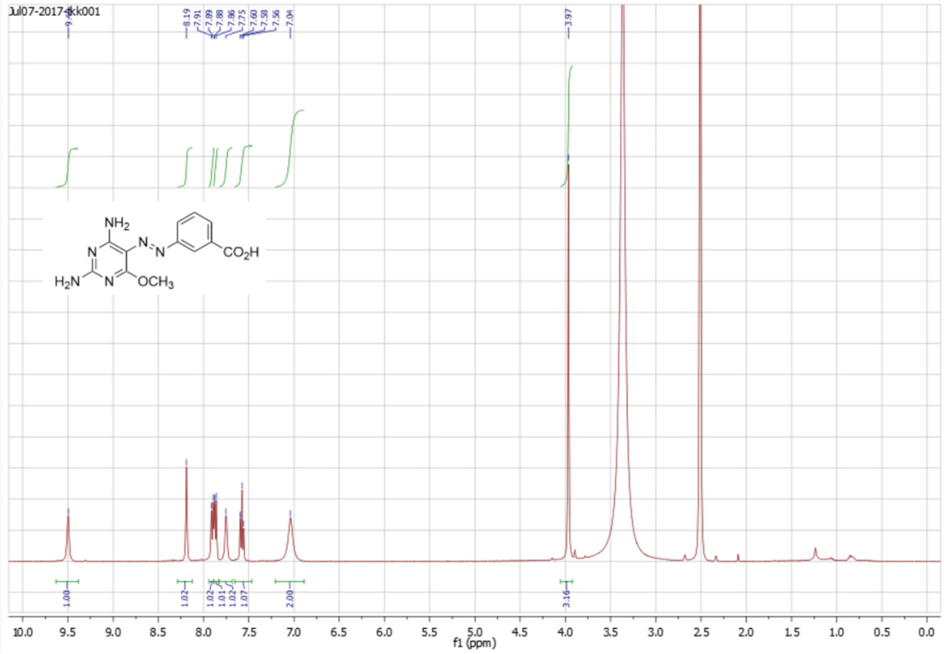

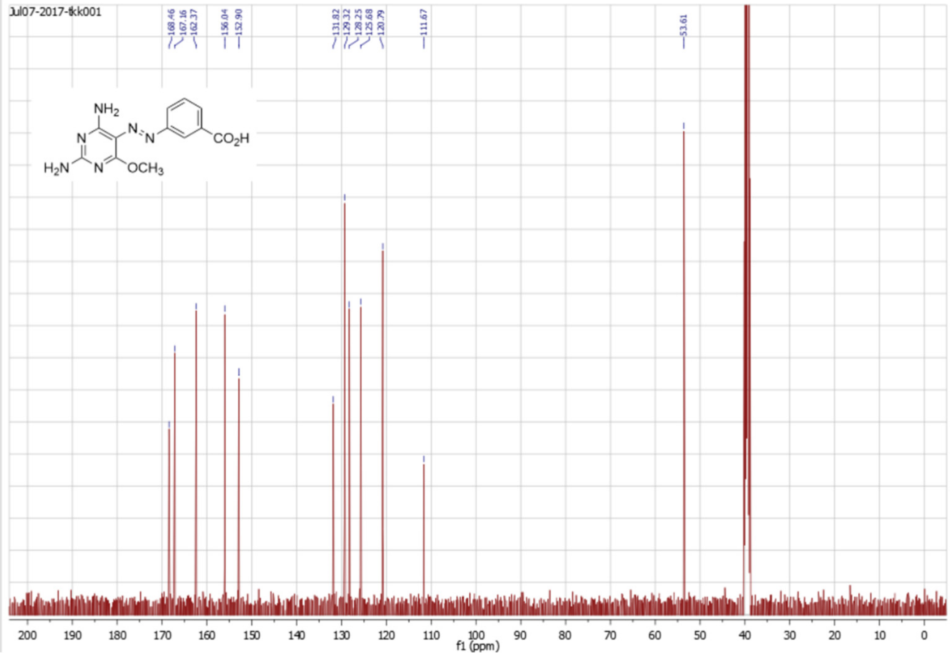
Jul07-2017-tkk001
168.46
167.16
162.37
156.04
152.90
131.82
129.32
128.25
125.68
120.79
111.67
53.61
NH2
N
N
H2N
N
OCH3
N
CO2H
f1 (ppm)

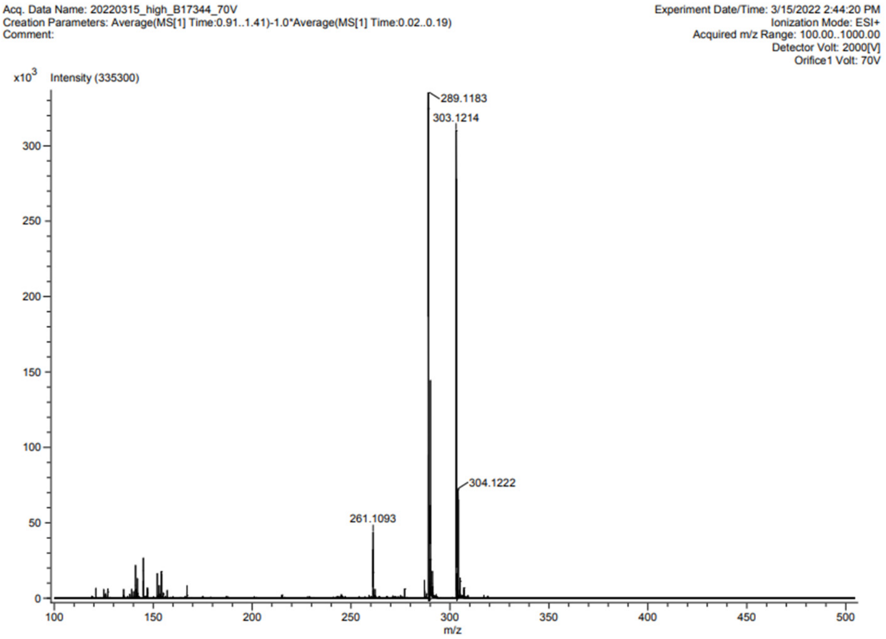
Acq. Data Name: 20220315_high_B17344_70V
Creation Parameters: Average(MS[1] Time:0.91..1.41)-1.0*Average(MS[1] Time:0.02..0.19)
Comment:
Experiment Date/Time: 3/15/2022 2:44:20 PM
Ionization Mode: ESI+
Acquired m/z Range: 100.00..1000.00
Detector Volt: 2000[V]
Orifice1 Volt: 70V
x10^3 Intensity (335300)
289.1183
303.1214
304.1222
261.1093
m/z

- ^{1}H (400 MHz, DMSO-d_6) and ^{13}C (400 MHz, DMSO-d_6) NMR spectra and DART-TOF HRMS spectrum of (*E*)-5-(phenyldiazenyl)pyrimidine-2,4,6-triamine (**4**)

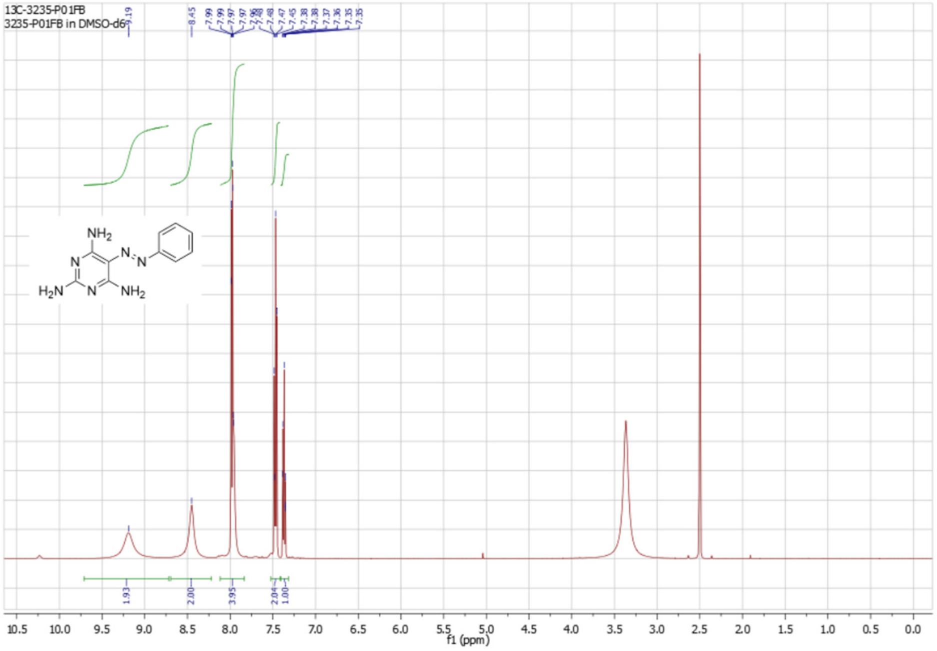

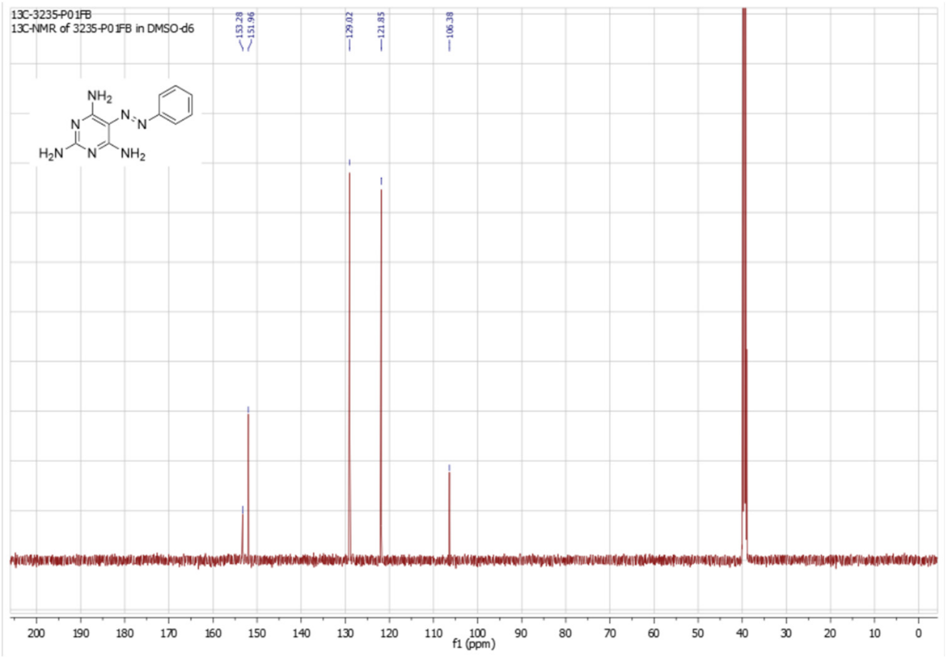

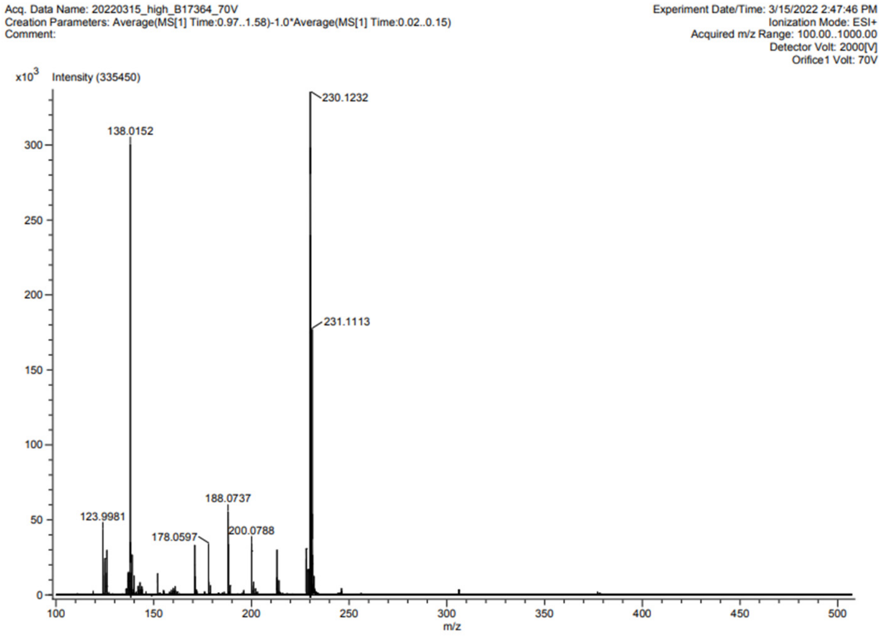

- ^{1}H (400 MHz, DMSO-d_6) and ^{13}C (101 MHz, DMSO-d_6) NMR spectra and DART-TOF HRMS spectrum of (*E*)-3-((2,4,6-triaminopyrimidin-5-yl)diazenyl)benzoic acid (**5**)

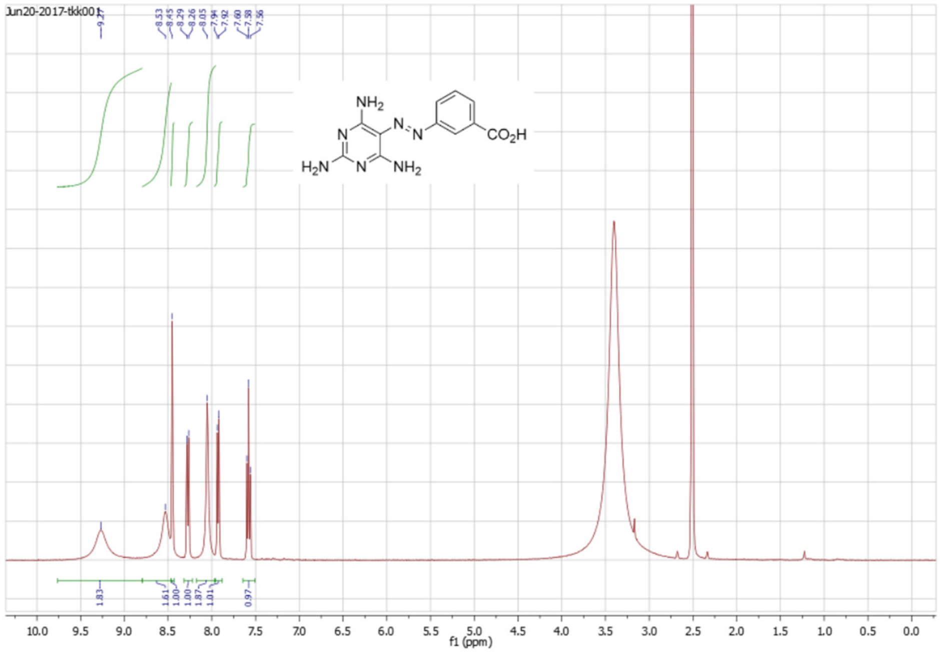

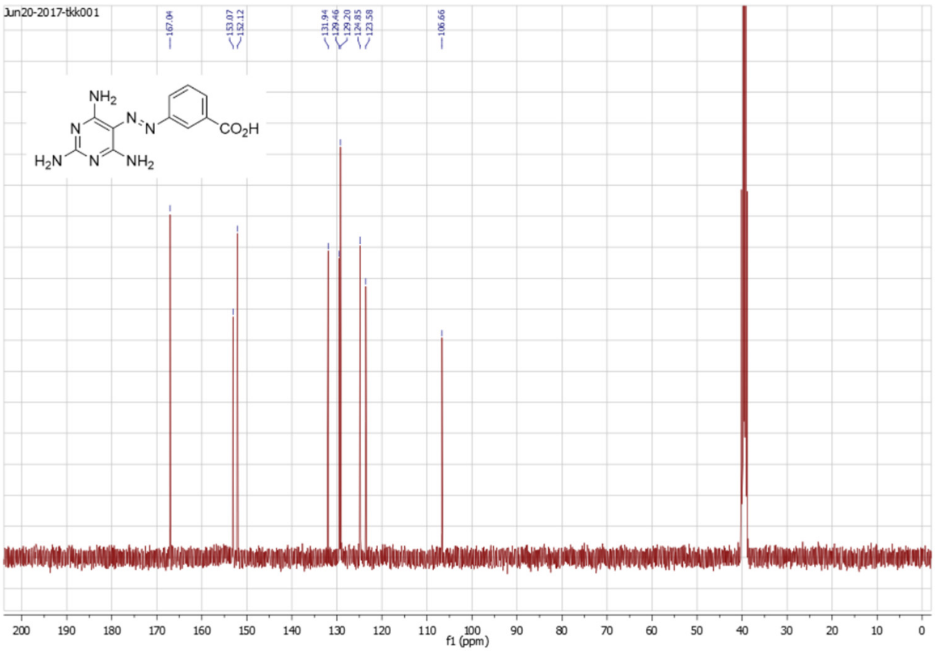

Jun20-2017-tkk001
167.04
153.07
152.12
131.94
129.46
129.20
124.85
123.58
106.66
NH2
N
N
N
H2N
N
NH2
CO2H
f1 (ppm)

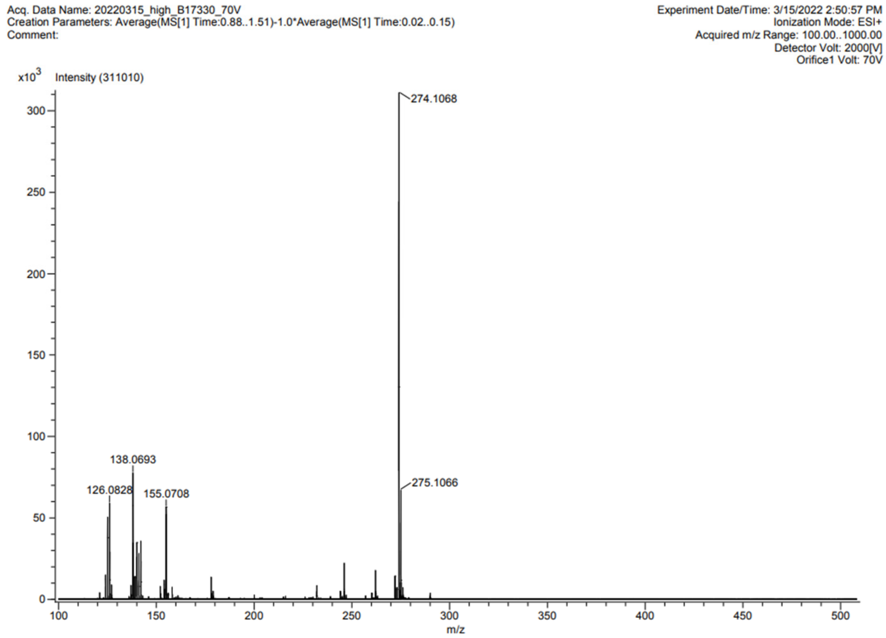

Acq. Data Name: 20220315_high_B17330_70V
Creation Parameters: Average(MS[1] Time:0.88..1.51)-1.0*Average(MS[1] Time:0.02..0.15)
Comment:
Experiment Date/Time: 3/15/2022 2:50:57 PM
Ionization Mode: ESI+
Acquired m/z Range: 100.00..1000.00
Detector Volt: 2000[V]
Orifice1 Volt: 70V
x10^3 Intensity (311010)
274.1068
138.0693
126.0828
155.0708
275.1066
m/z

- ^{1}H (500 MHz, DMSO-d_6) and ^{13}C (126 MHz, DMSO-d_6) NMR spectra and DART-TOF HRMS spectrum of 4-(4-((2-amino-4-methyl-6-oxo-1,6-dihydropyrimidin-5-yl)methyl)-1H-1,2,3-triazol-1-yl)benzoic acid (**6**)

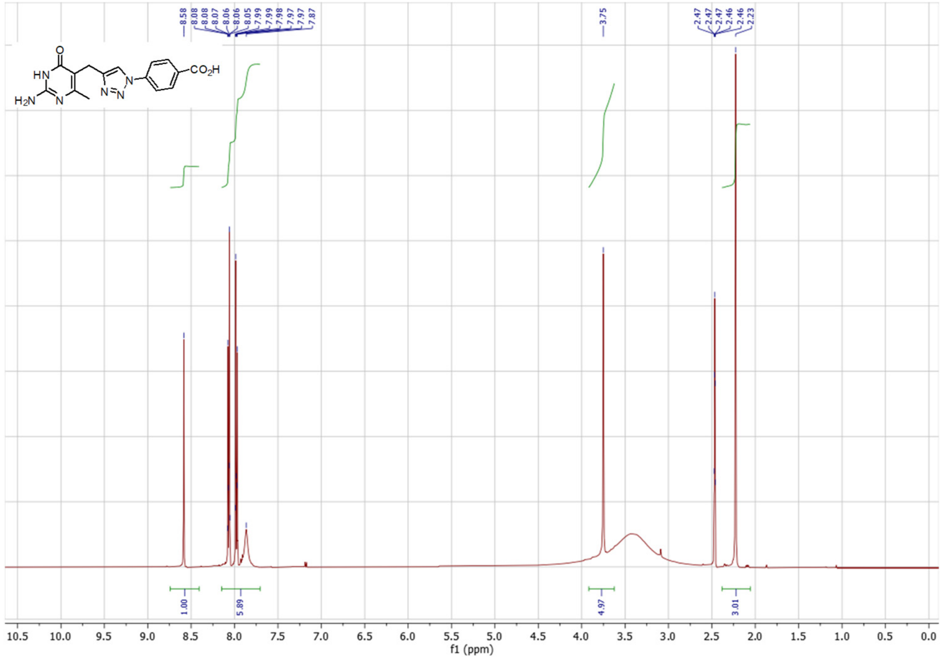

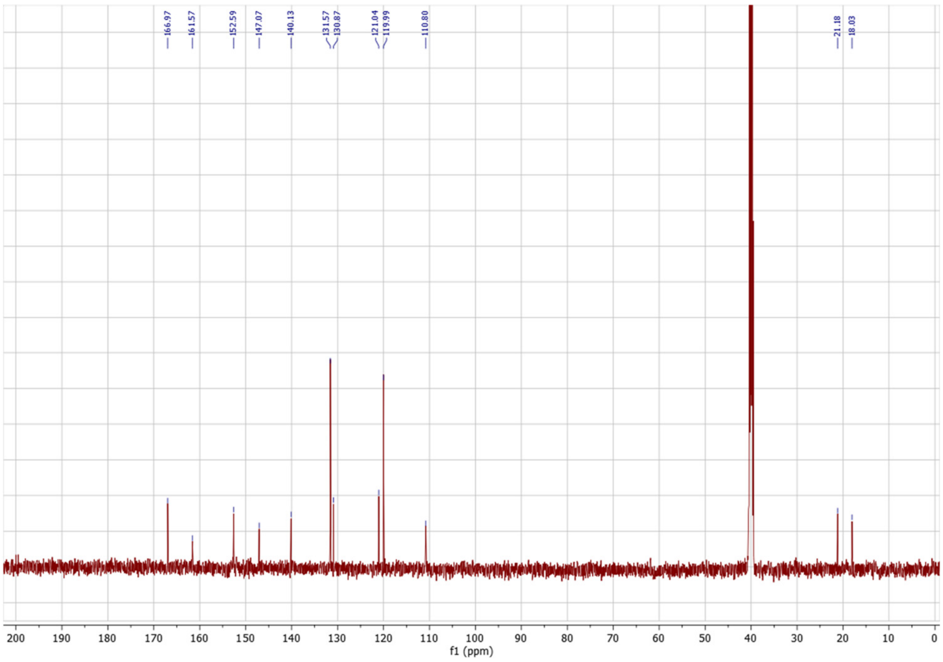

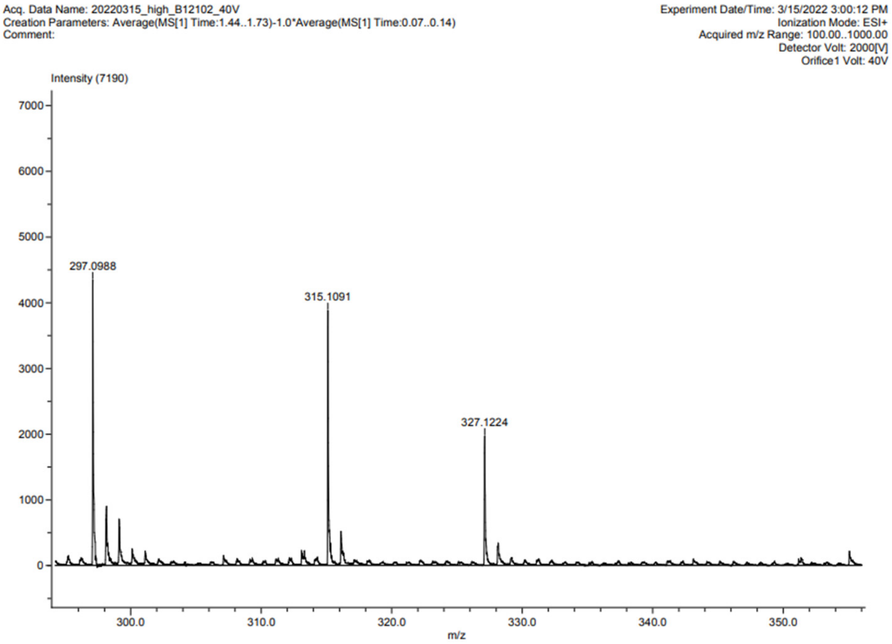

- ^1H (500 MHz, DMSO-d_6) and ^{13}C (126 MHz, DMSO-d_6) NMR spectra and DART-TOF HRMS spectrum of 3-(4-((2-amino-4-methyl-6-oxo-1,6-dihydropyrimidin-5-yl)methyl)-1H-1,2,3-triazol-1-yl)benzoic acid (**7**)

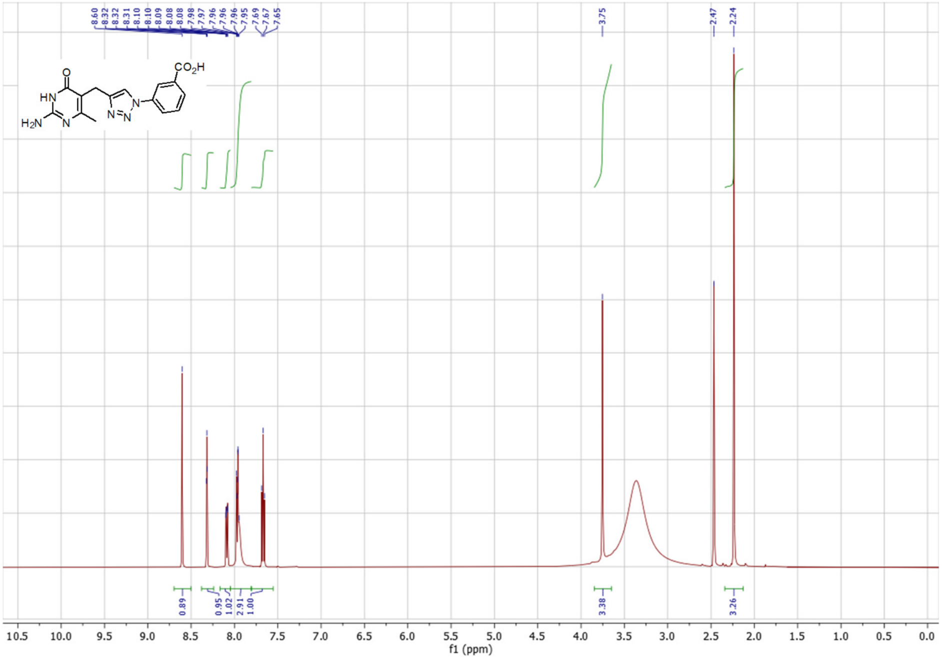

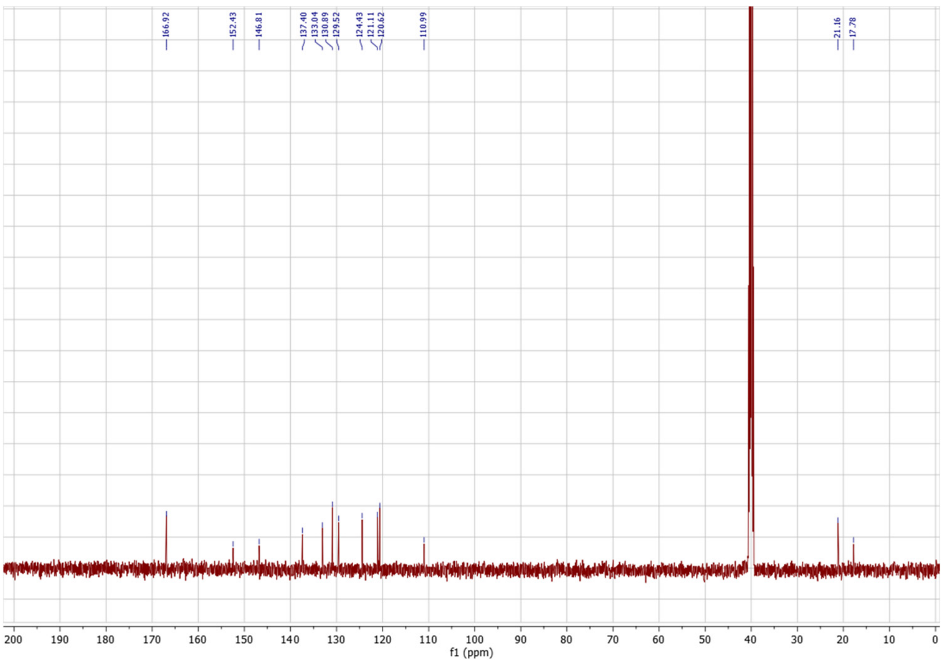
166.92
152.43
146.81
137.40
133.04
130.89
129.52
124.43
121.11
120.62
110.99
21.16
17.78
f1 (ppm)

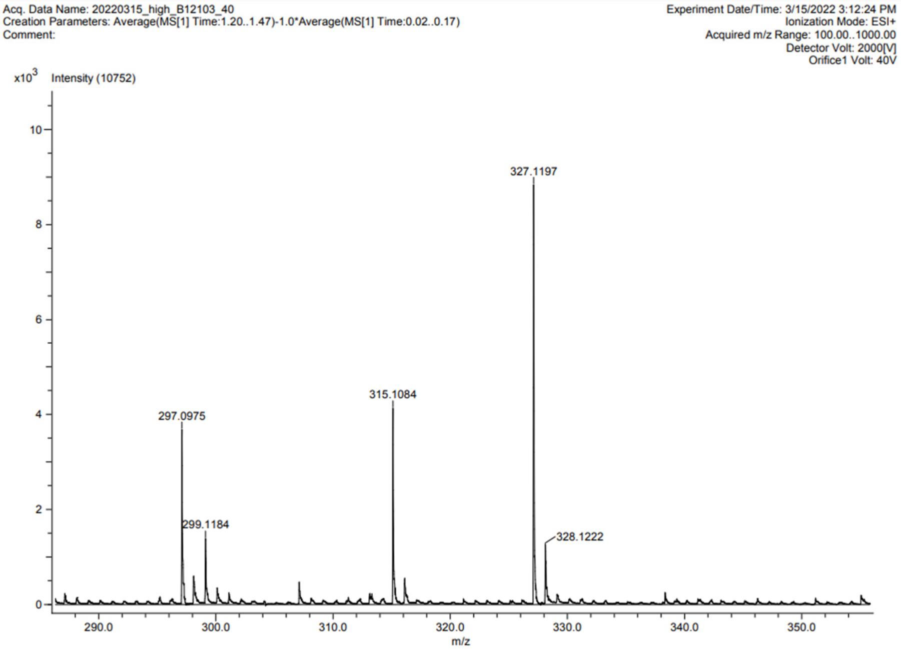
Acq. Data Name: 20220315_high_B12103_40
Creation Parameters: Average(MS[1] Time:1.20..1.47)-1.0*Average(MS[1] Time:0.02..0.17)
Comment:
Experiment Date/Time: 3/15/2022 3:12:24 PM
Ionization Mode: ESI+
Acquired m/z Range: 100.00..1000.00
Detector Volt: 2000[V]
Orifice1 Volt: 40V
x10^3 Intensity (10752)
327.1197
315.1084
297.0975
299.1184
328.1222
m/z

- ^1H (500 MHz, DMSO-d_6) and ^{13}C (126 MHz, DMSO-d_6) NMR spectra and DART-TOF HRMS spectrum of 4-(4-((2-amino-4-ethyl-6-oxo-1,6-dihydropyrimidin-5-yl)methyl)-1H-1,2,3-triazol-1-yl)benzoic acid (**8**)

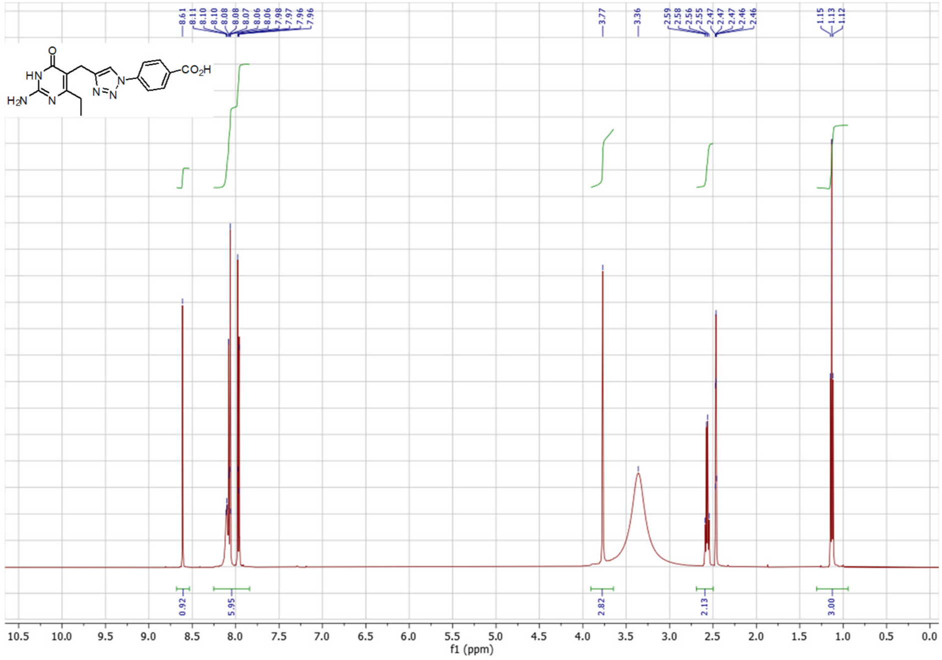

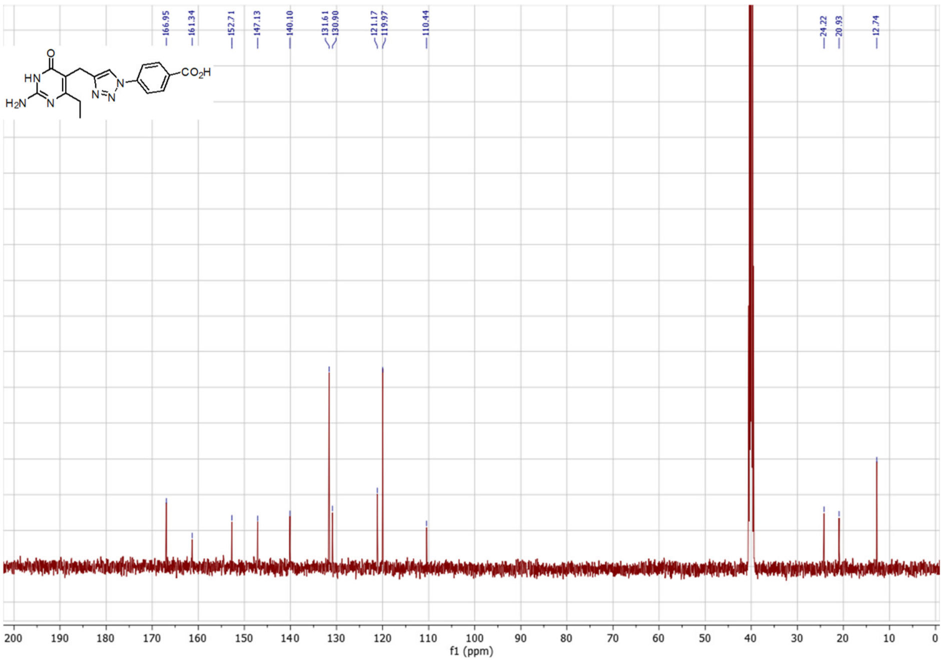

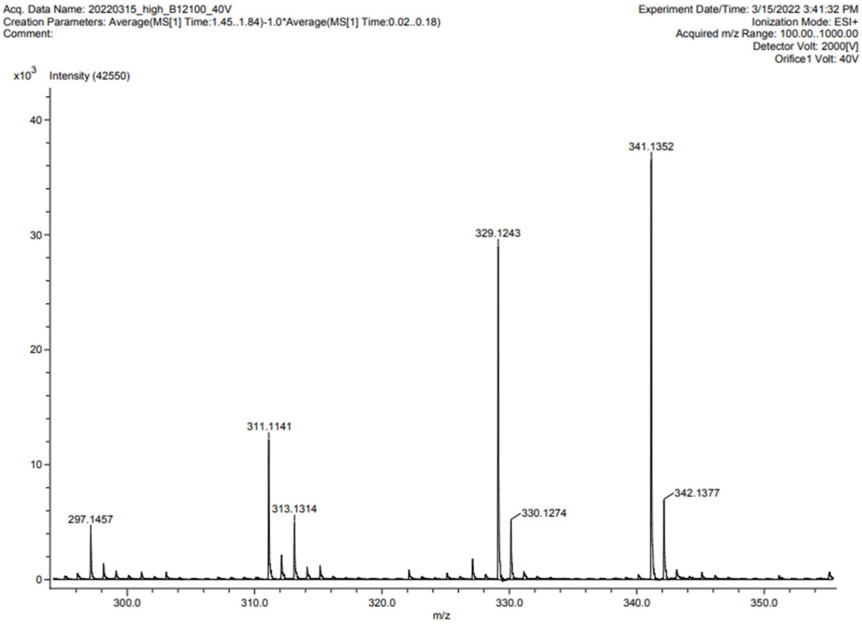

- ^{1}H (500 MHz, DMSO-d_6) and ^{13}C (126 MHz, DMSO-d_6) NMR spectra and DART-TOF HRMS spectrum of 3-(4-((2-amino-4-ethyl-6-oxo-1,6-dihydropyrimidin-5-yl)methyl)-1H-1,2,3-triazol-1-yl)benzoic acid (**9**)

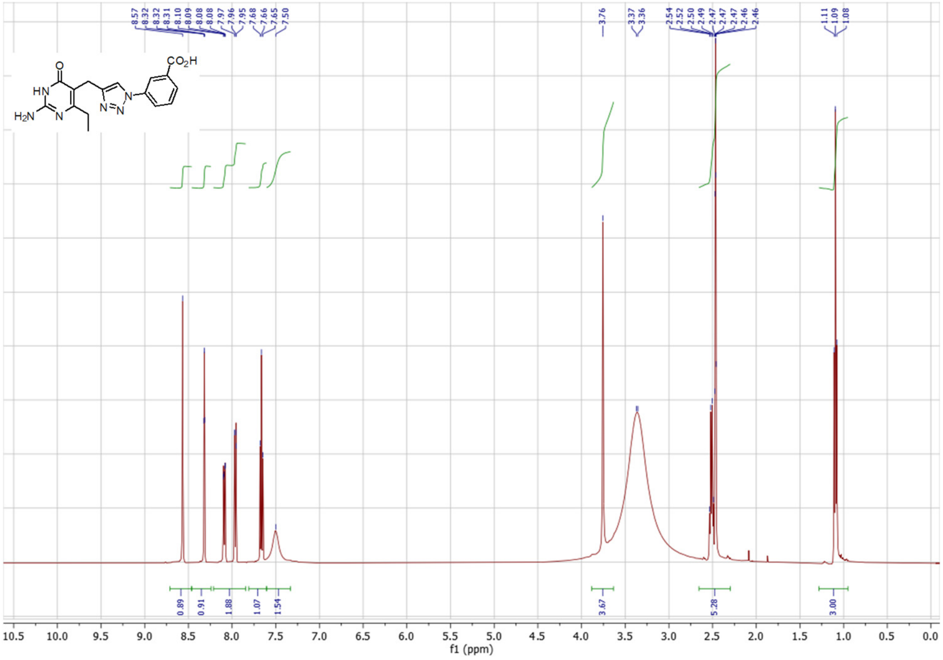

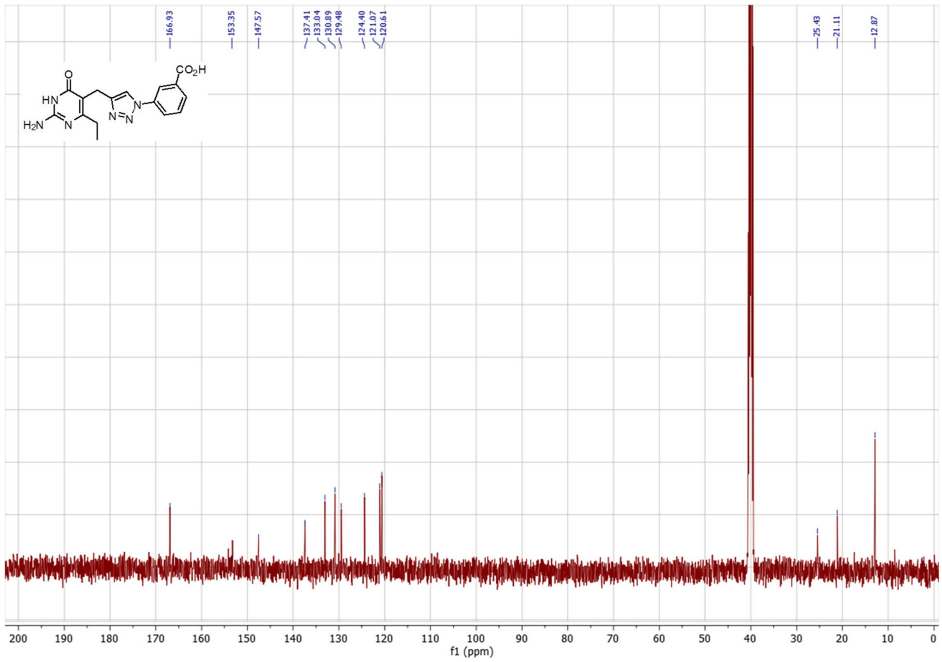
166.93
153.35
147.57
137.41
133.04
130.89
129.48
124.40
121.07
120.61
25.43
21.11
12.87
f1 (ppm)

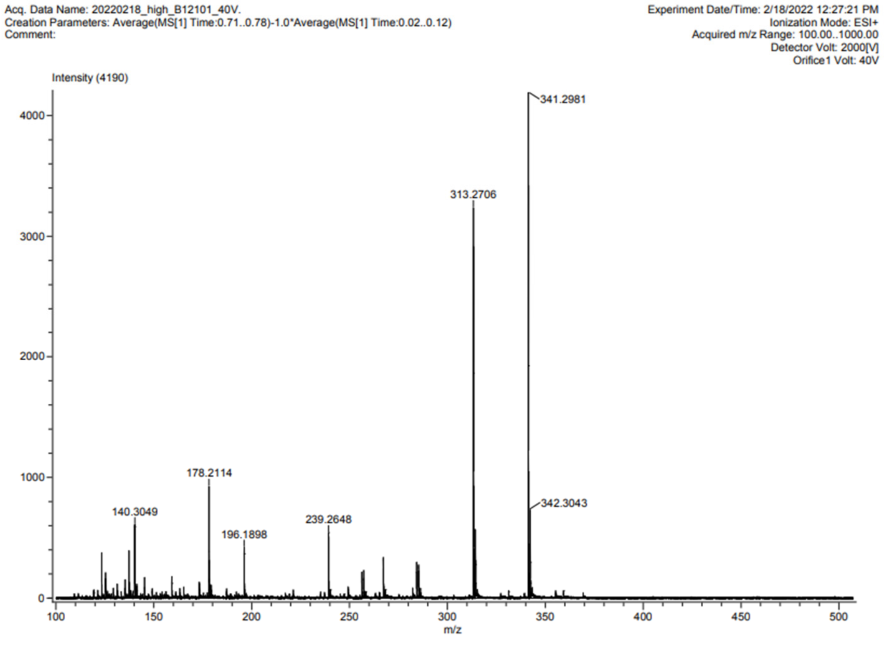
Acq. Data Name: 20220218_high_B12101_40V.
Creation Parameters: Average(MS[1] Time:0.71..0.78)-1.0*Average(MS[1] Time:0.02..0.12)
Comment:
Experiment Date/Time: 2/18/2022 12:27:21 PM
Ionization Mode: ESI+
Acquired m/z Range: 100.00..1000.00
Detector Volt: 2000[V]
Orifice1 Volt: 40V
Intensity (4190)
341.2981
313.2706
178.2114
140.3049
196.1898
239.2648
342.3043
m/z

- ^{1}H (400 MHz, DMSO-d_6) and ^{13}C (101 MHz, DMSO-d_6) NMR spectra and DART-TOF HRMS spectrum of 2-amino-6-ethyl-5-((1-(3-(nitro)phenyl)-1H-1,2,3-triazol-4-yl)methyl)pyrimidin-4(3*H*)-one (**10**)

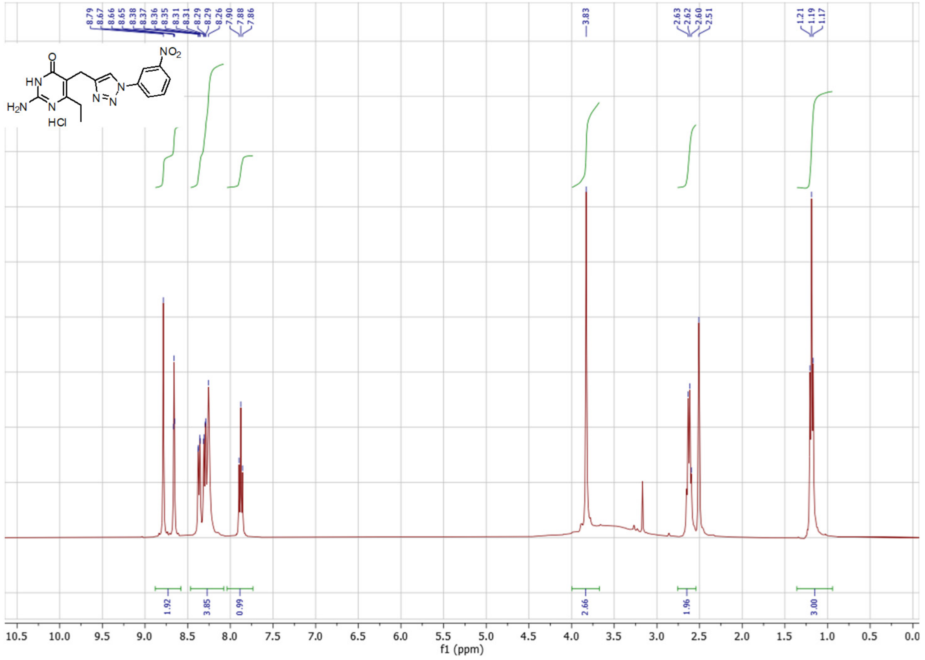

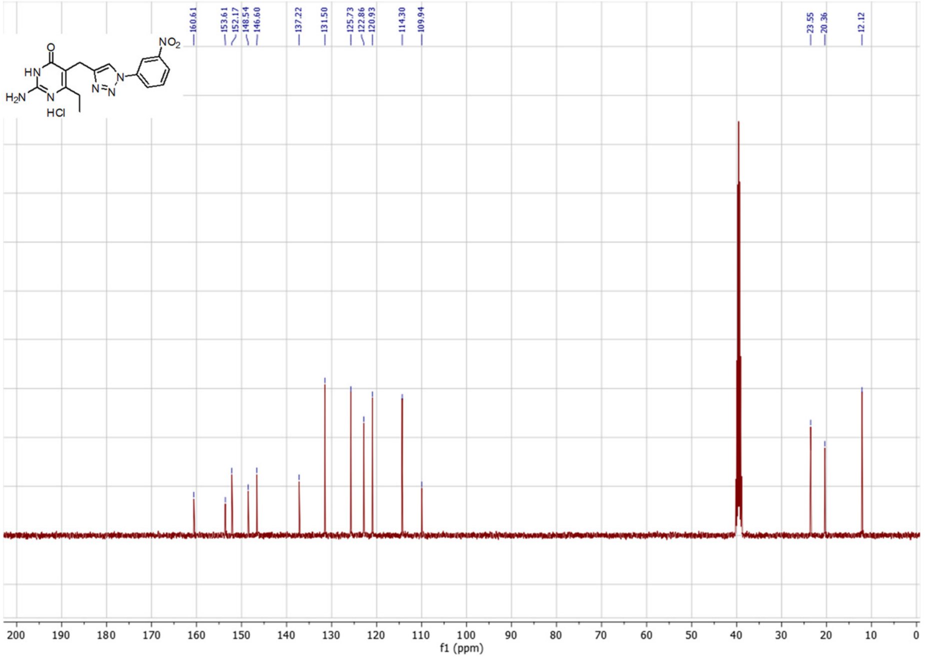

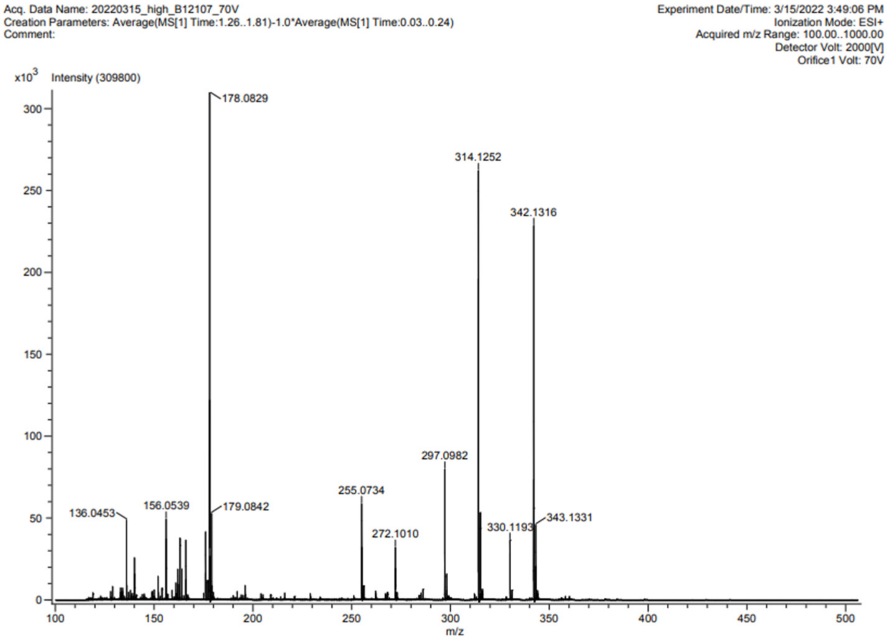

- ^{1}H (400 MHz, DMSO-d_6) and ^{13}C (101 MHz, DMSO-d_6) NMR spectra and DART-TOF HRMS spectrum of 2-amino-6-ethyl-5-(2-(1-(4-(carboxy)phenyl)-1H-1,2,3-triazol-4-yl)ethyl)pyrimidin-4(3H)-one (**11**)

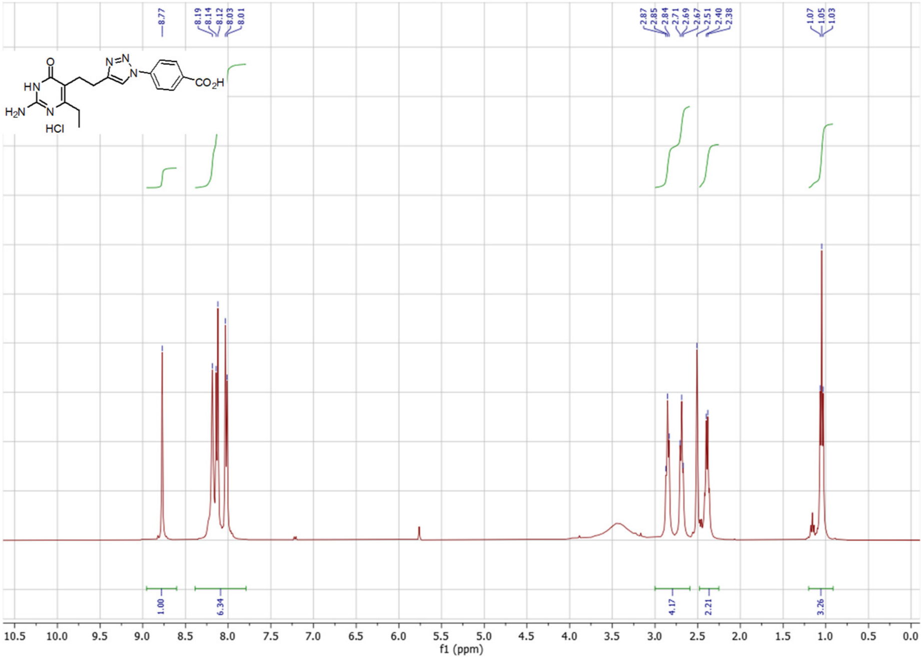

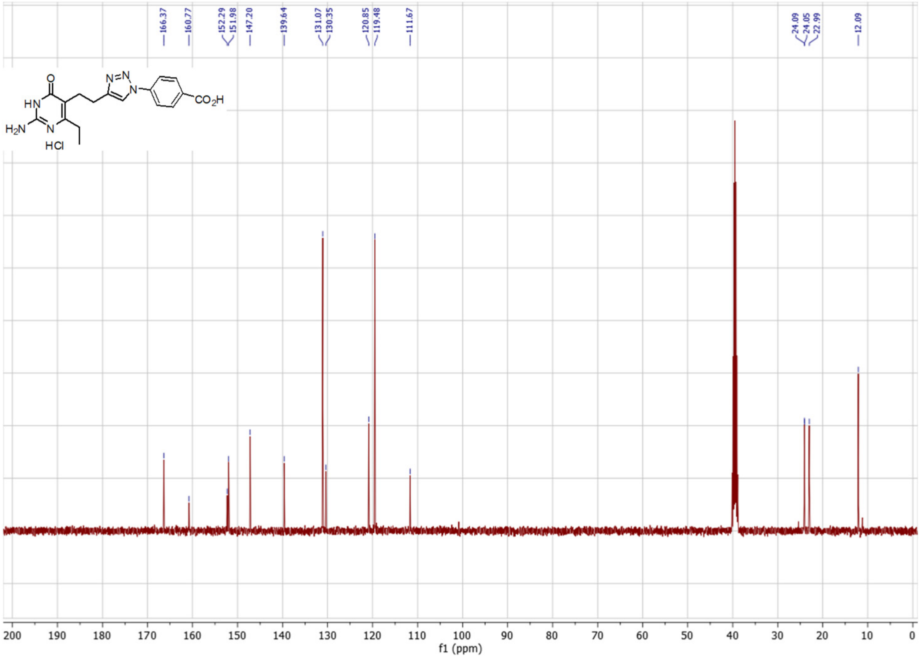
166.37
160.77
152.29
151.98
147.20
139.64
131.07
130.35
120.85
119.48
111.67
24.09
24.05
22.99
12.09
HN
O
N
H2N
HCl
N=N
N
CO2H
200 190 180 170 160 150 140 130 120 110 100 90 80 70 60 50 40 30 20 10 0
f1 (ppm)

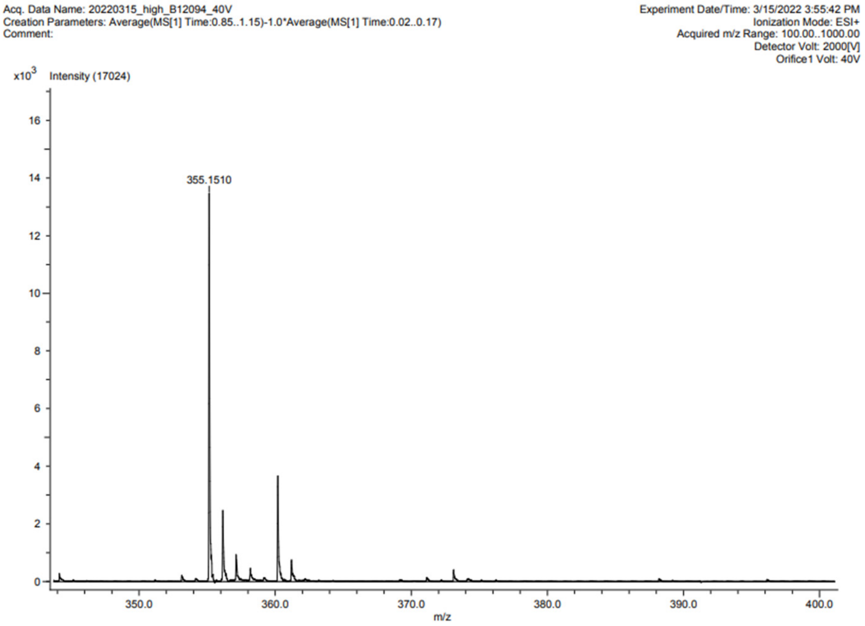
Acq. Data Name: 20220315_high_B12094_40V
Creation Parameters: Average(MS[1] Time:0.85..1.15)-1.0*Average(MS[1] Time:0.02..0.17)
Comment:
Experiment Date/Time: 3/15/2022 3:55:42 PM
Ionization Mode: ESI+
Acquired m/z Range: 100.00..1000.00
Detector Volt: 2000[V]
Orifice1 Volt: 40V
x10^3 Intensity (17024)
355.1510
0 2 4 6 8 10 12 14 16
350.0 360.0 370.0 380.0 390.0 400.0
m/z

- ^{1}H (400 MHz, DMSO-d_6) and ^{13}C (101 MHz, DMSO-d_6) NMR spectra and DART-TOF HRMS spectrum of 2-amino-6-ethyl-5-(2-(1-(3-(carboxy)phenyl)-1H-1,2,3-triazol-4-yl)ethyl)pyrimidin-4(3H)-one (**12**)

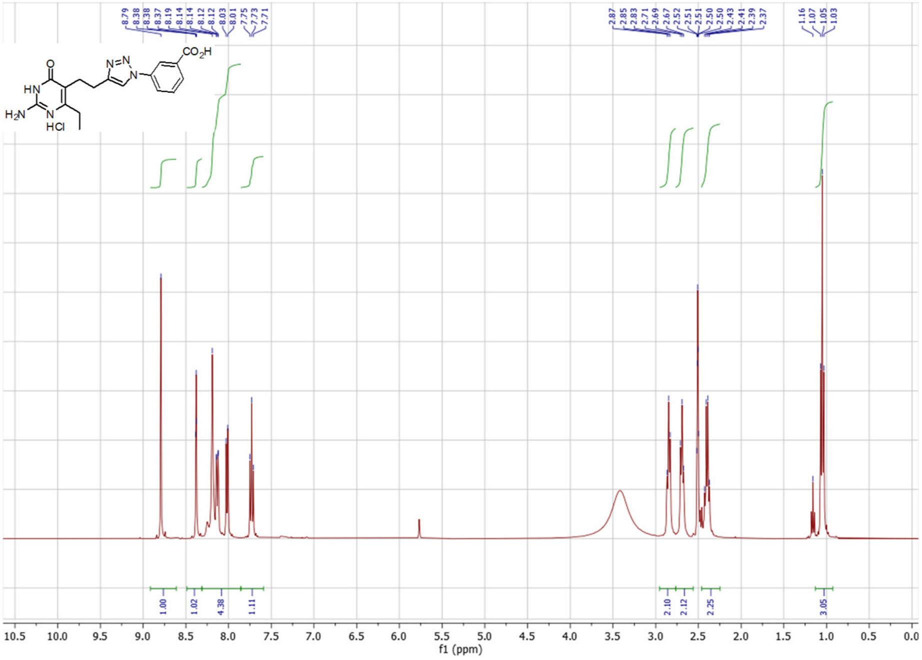

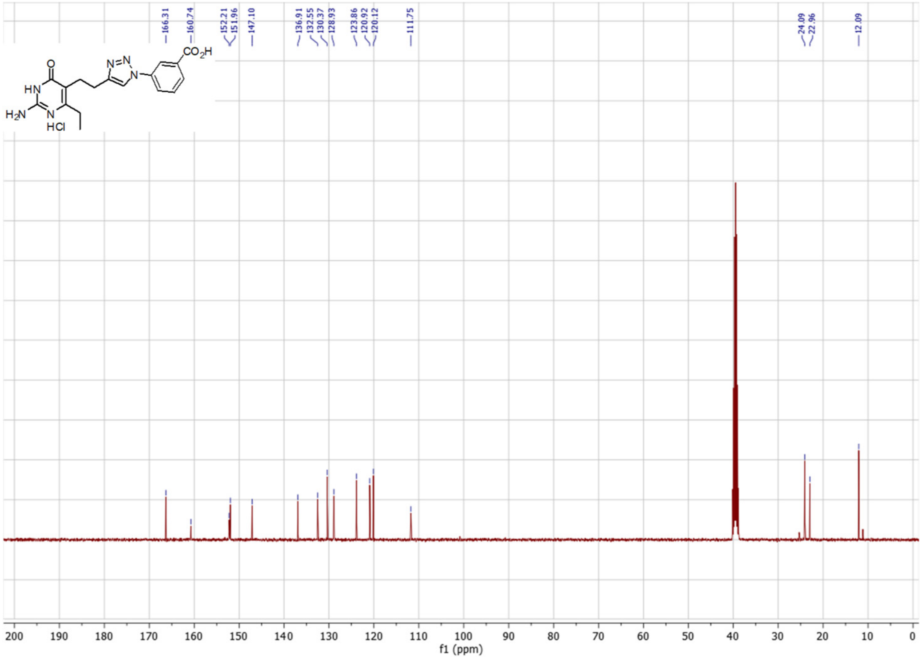

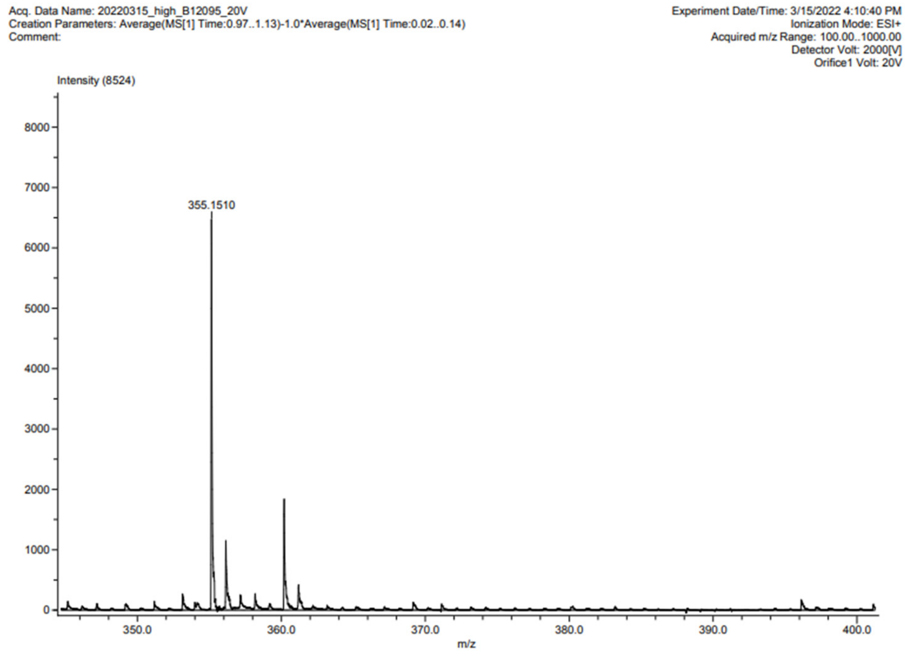

- ^{1}H (400 MHz, DMSO-d_6) and ^{13}C (101 MHz, DMSO-d_6) NMR spectra and DART-TOF HRMS spectrum of 2-amino-6-ethyl-5-(3-(1-(4-(carboxy)phenyl)-1H-1,2,3-triazol-4-yl)propyl)pyrimidin-4(3H)-one (**13**)

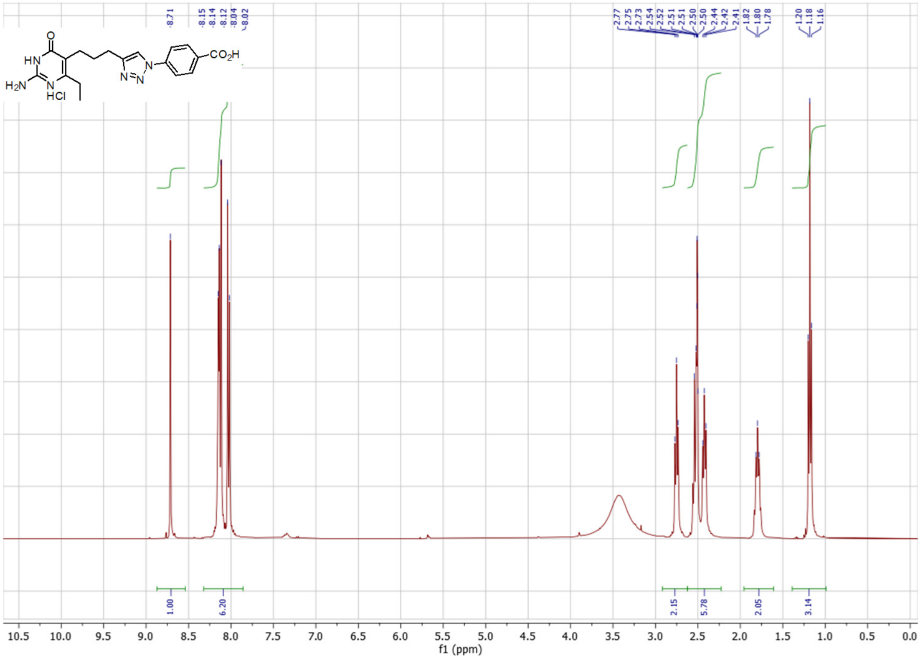

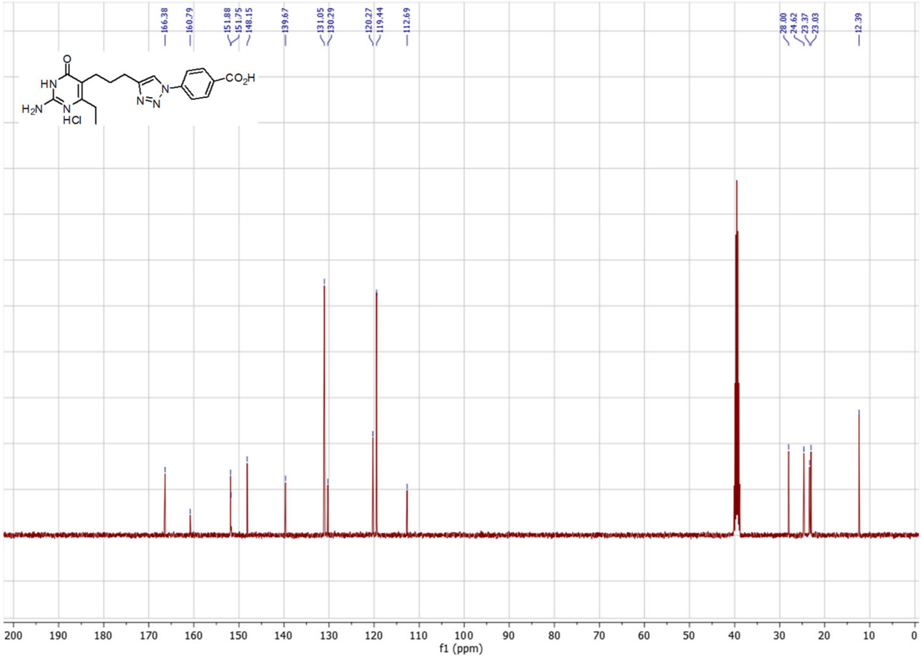
166.38
160.79
151.88
151.75
148.15
139.67
131.05
130.29
120.27
119.44
112.69
28.00
24.62
23.37
23.03
12.39
HN
H2N
N
HCl
O
N
N
N
CO2H
f1 (ppm)

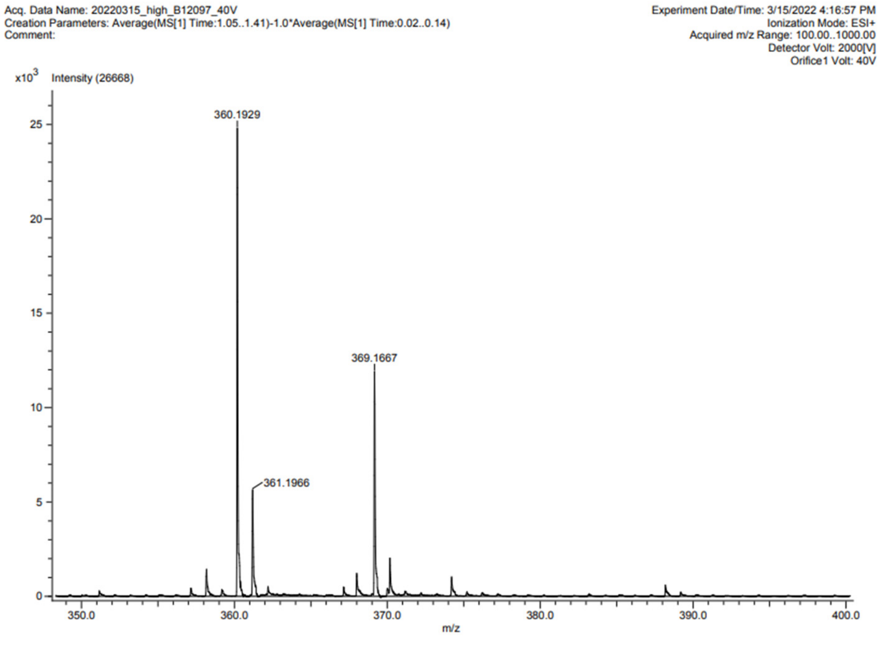
Acq. Data Name: 20220315_high_B12097_40V
Creation Parameters: Average(MS[1] Time:1.05..1.41)-1.0*Average(MS[1] Time:0.02..0.14)
Comment:
Experiment Date/Time: 3/15/2022 4:16:57 PM
Ionization Mode: ESI+
Acquired m/z Range: 100.00..1000.00
Detector Volt: 2000[V]
Orifice1 Volt: 40V
x10^3 Intensity (26668)
360.1929
361.1966
369.1667
m/z

- ^{1}H (400 MHz, DMSO-d_6) and ^{13}C (100 MHz, DMSO-d_6) NMR spectra and DART-TOF HRMS spectrum of 2-amino-6-ethyl-5-(3-(1-(3-(carboxy)phenyl)-1H-1,2,3-triazol-4-yl)propyl)pyrimidin-4(3H)-one (**14**)

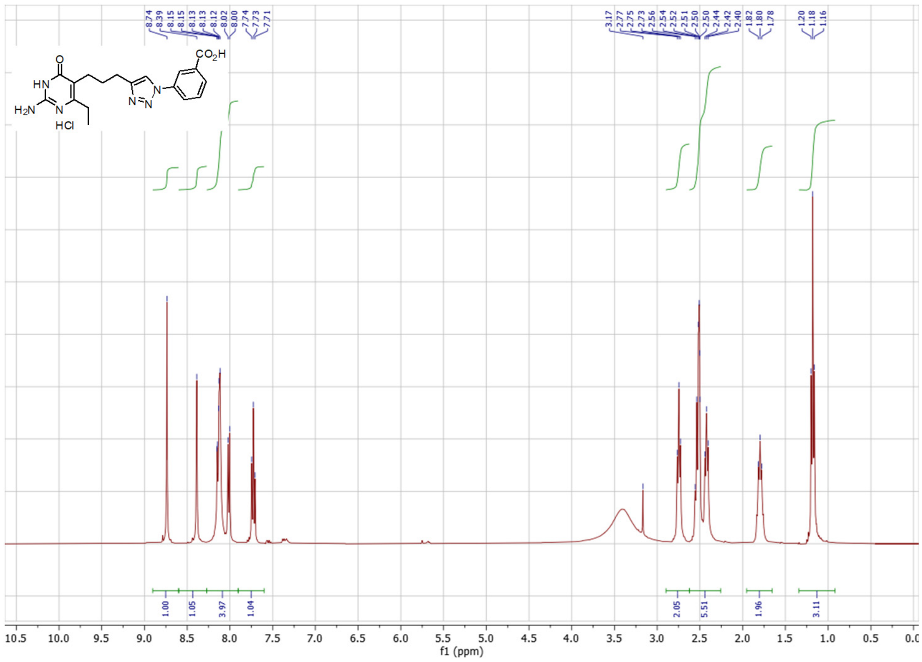

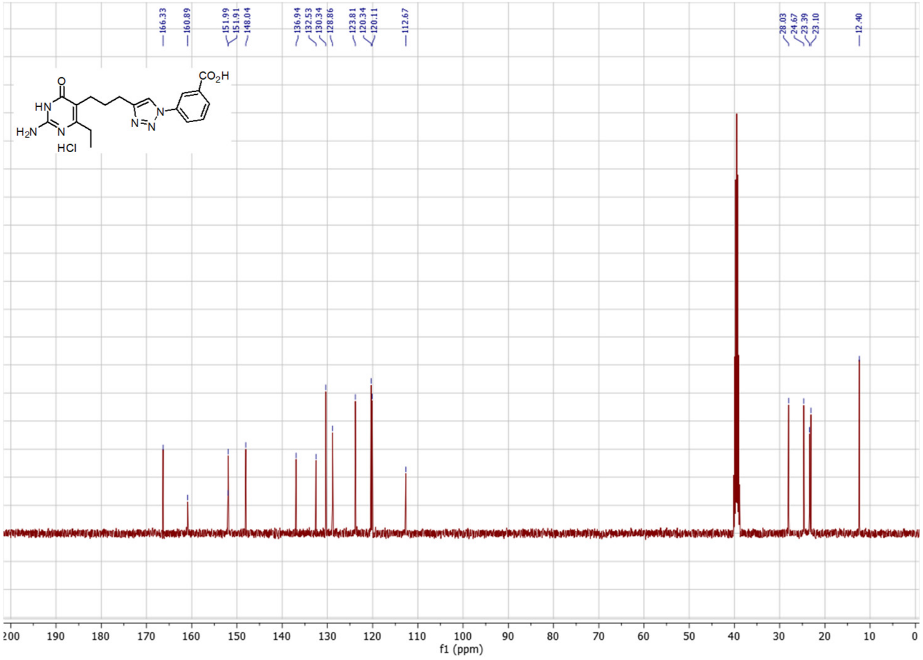

Acq. Data Name: 20220315_high_B12098_40V
Creation Parameters: Average(MS[1] Time:1.07..1.68)-1.0*Average(MS[1] Time:0.02..0.19)
Comment:

Experiment Date/Time: 3/15/2022 4:27:35 PM
Ionization Mode: ESI+
Acquired m/z Range: 100.00..1000.00
Detector Volt: 2000[V]
Orifice1 Volt: 40V

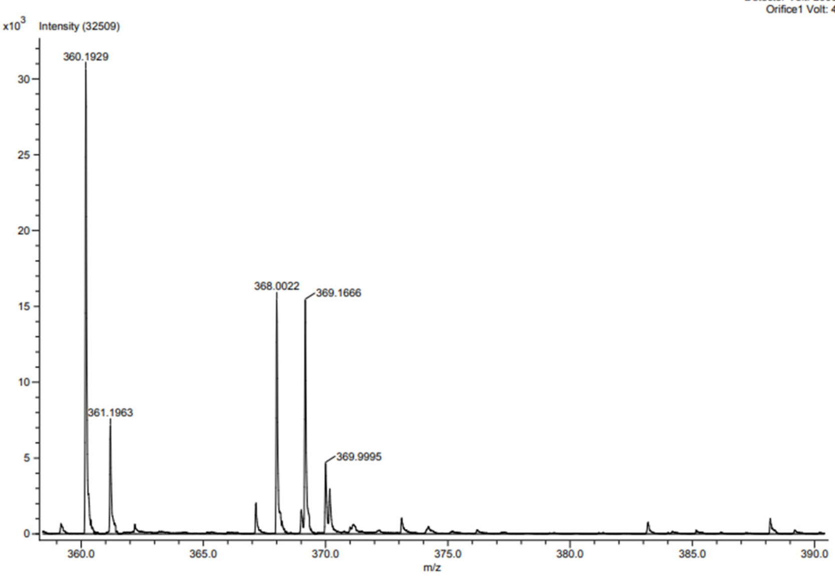

Figure S2: Protein sequencing result for *Pf*HPPK-GFP. Peptides observed by LC-MS/MS appear in blue, post-translational modifications appear as mentioned in the legend.

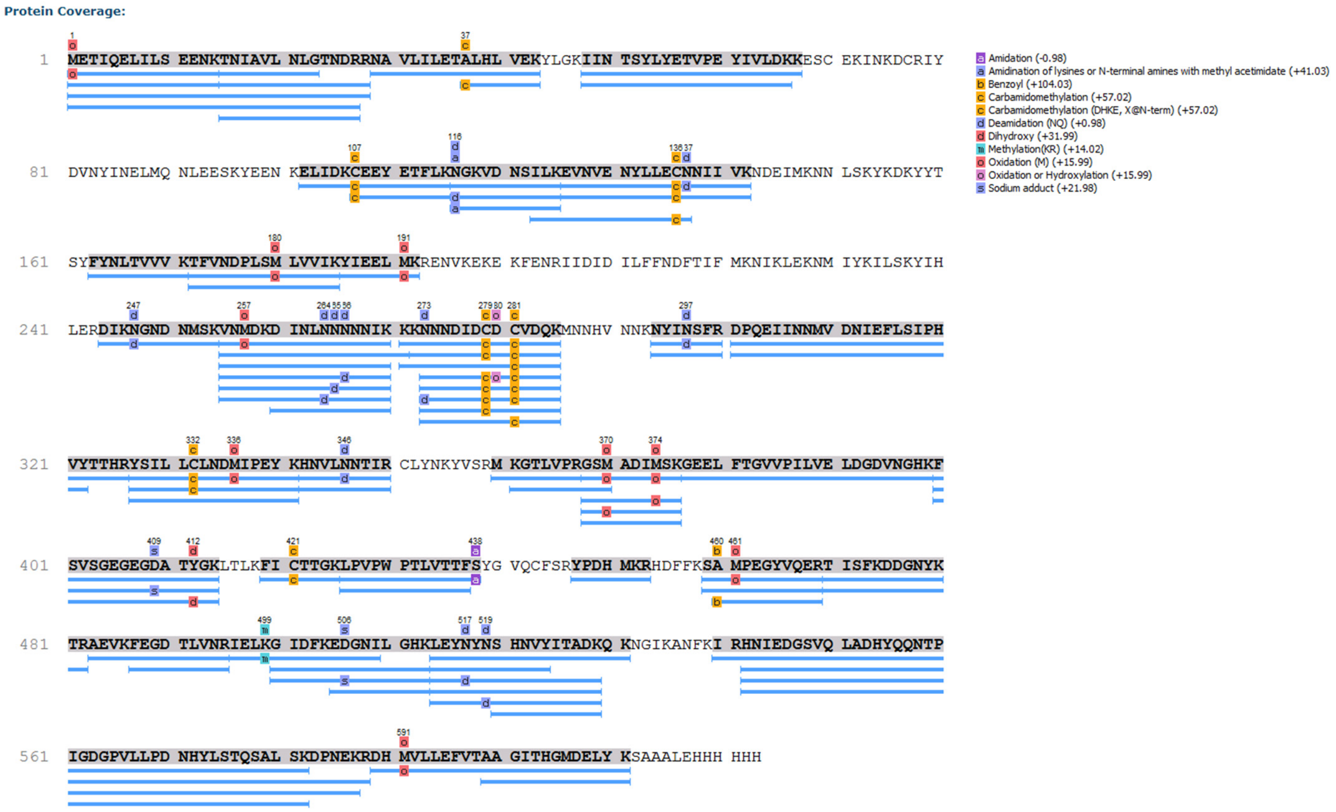

Figure S3: GFP melting temperature in the presence of various concentrations of ATP.

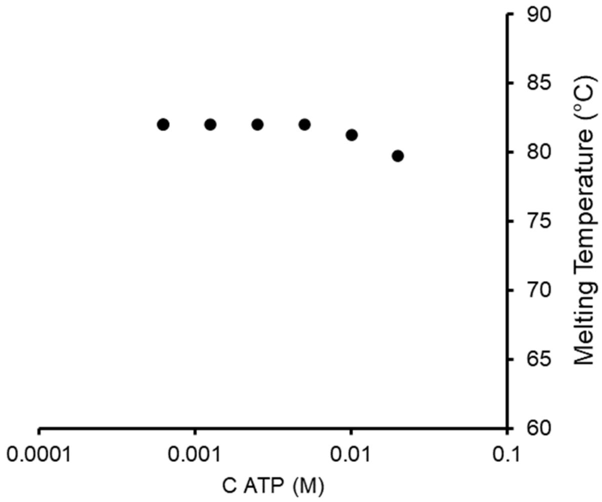

Figure S4: DSF curves obtained for HPPK-GFP and GFP proteins.

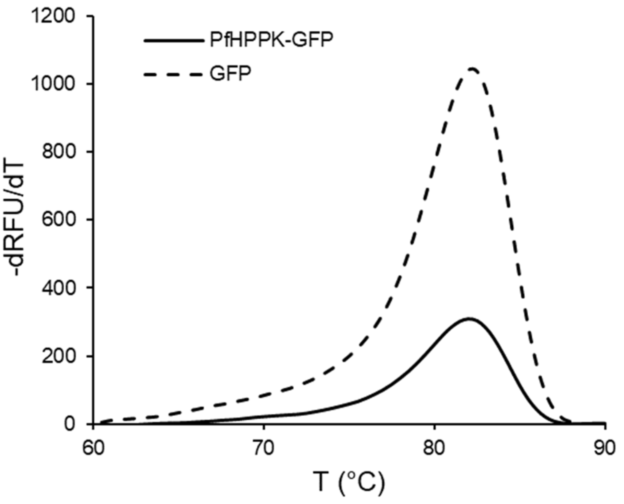

Table S1. Molecular docking scores obtained from antifolate library virtual screening. Hit compounds appear in bold.

Code	Smiles	Docking score
B293	CN(C(N=C(N)N1)=C2C1=O)N=C(CC(N)=O)C2=O	-8.85
B266	CN(C(N=C(N)N1)=C2C1=O)N=C(C(O)=O)C2=O	-8.64
**13**	CCC(N=C(N)N1)=C(CCCc2cn(-c(cc3)ccc3C(O)=O)nn2)C1=O.Cl	-8.63
**14**	CCC(N=C(N)N1)=C(CCCc2cn(-c3cc(C(O)=O)ccc3)nn2)C1=O.Cl	-8.48
B099	CCC(N=C(N)N1)=C(CCCc2cn(-c(cc3)ccc3[N+]([O-])=O)nn2)C1=O.Cl	-8.48
B109	CCC(N=C(N)N1)=C(CCCc2cn(-c3cccc([N+]([O-])=O)c3)nn2)C1=O.Cl	-8.43
**10**	CCC(N=C(N)N1)=C(Cc2cn(-c3cccc([N+]([O-])=O)c3)nn2)C1=O.Cl	-8.38
B244	CC(C(O)=O)C(C1=O)=NN(C)C(N=C(N)N2)=C1C2=O	-8.34
**11**	CCC(N=C(N)N1)=C(CCc2cn(-c(cc3)ccc3C(O)=O)nn2)C1=O.Cl	-8.30
B105	CNC(N=C(N)NC1=O)=C1[N+]([O-])=O	-8.17
B324	CCc1nc(N)nc(N)c1OCCCOc(cc1)cc2c1c(C([O-])=O)cn2CCCOc(cc1)cc2c1c(C([O-])=O)c[nH]2	-8.15
B453	NC(N1)=Nc2nn(-c3cccc(C(NCc(cc4)ccc4Cl)=O)c3)nc2C1=O	-8.09
B267	CC(C(c(nc12)cnc1N=C(N)NC2=O)O)O	-8.07
B302	CCc1nc(N)nc(N)c1OCCCOc1cccc(C(N[C@@H](CCC([O-])=O)C([O-])=O)=O)c1	-7.96
B265	CN(C(N=C(N)N1)=C2C1=O)N=C(CC(O)=O)C2=O	-7.91
**12**	CCC(N=C(N)N1)=C(CCc2cn(-c3cc(C(O)=O)ccc3)nn2)C1=O.Cl	-7.91
B106	CCC(N=C(N)N1)=C(Cc2cn(-c(cc3)ccc3[N+]([O-])=O)nn2)C1=O.Cl	-7.88
B108	CCC(N=C(N)N1)=C(CCc2cn(-c3cccc([N+]([O-])=O)c3)nn2)C1=O.Cl	-7.77
B387	NC(NC1=O)=Nc([nH]2)c1nc2SCC(c1ccccc1)=O	-7.71
B388	NC(NC1=O)=Nc([nH]2)c1nc2SCc1ccccc1	-7.42

B291	NC(N1)=NC(NN=C(CC(O)=O)C2=O)=C2C1=O	-7.39
B367	Nc1nc(N)nc(O)c1/N=N/c(cc1)ccc1Cl	-7.37
B332	Nc(nc(N)nc1O)c1/N=N/c1ccccc1	-7.35
B365	Nc1nc(N)nc(O)c1/N=N/c(cc1)ccc1F	-7.33
B366	Cc(cc1)ccc1/N=N/c1c(N)nc(N)nc1O	-7.26
B294	CN(C(N=C(N)N1)=C2C1=O)N=C(CCO)C2=O	-7.16
B369	Nc1nc(N)nc(O)c1/N=N/c1cccc(Cl)c1	-7.14
B370	Nc1nc(N)nc(O)c1/N=N/c1cccc(C(F)(F)F)c1	-7.11
**6**	CC(N=C(N)N1)=C(Cc2cn(-c(cc3)ccc3C(O)=O)nn2)C1=O.Cl	-6.86
B346	COC(c1cc(/N=N/c(c(N)nc(N)n2)c2O)ccc1)=O	-6.86
B256	NC(N=C(N)NC1=O)=C1[N+]([O-])=O	-6.85
B035	CCc1nc(N)nc(N)c1OCCCc1cn(Cc(cc2)ccc2C(O)=O)nn1.Cl	-6.72
B368	Nc1nc(N)nc(O)c1/N=N/c(cccc1)c1C(O)=O	-6.70
B065	CCc1nc(N)nc(N)c1OCCc1cn(-c2cccc(C(O)=O)c2)nn1.Cl	-6.52
B036	CCc1nc(N)nc(N)c1OCCCc1cn(-c(cc2)ccc2C(O)=O)nn1.Cl	-6.44
B264	Nc1nc(N)nc(N)c1[N+]([O-])=O	-6.38
B217	CNc(nc(N)nc1N)c1[N+]([O-])=O	-6.10
B218	COC(c(cc1)ccc1NCc1nc2c(N)nc(N)nc2nc1)=O	-6.09
**1**	COc(nc(N)nc1N)c1/N=N/c1cccc([N+]([O-])=O)c1	-6.03
B032	CCc1nc(N)nc(N)c1OCc1cn(-c2cccc(C(O)=O)c2)nn1.Cl	-5.98
B220	Nc1c2nc(CNc3cccc(Cl)c3)cnc2nc(N)n1	-5.94
B341	Nc(nc(N)nc1Cl)c1/N=N/c1cc([N+]([O-])=O)ccc1	-5.83
B050	CCc1nc(N)nc(N)c1OCCc1cn(-c(cc2)ccc2C(O)=O)nn1.Cl	-5.71
B245	Nc1c2nc[nH]c2cnn1	-5.67
**9**	CCC(N=C(N)N1)=C(Cc2cn(-c3cc(C(O)=O)ccc3)nn2)C1=O.Cl	-5.64
B249	Nc1c2[nH]cnc2c(N)nn1	-5.64
**7**	CC(N=C(N)N1)=C(Cc2cn(-c3cc(C(O)=O)ccc3)nn2)C1=O.Cl	-5.51
B066	CCc1nc(N)nc(N)c1OCCCc1cn(-c2cccc(C(O)=O)c2)nn1.Cl	-5.46
**3**	COc(nc(N)nc1N)c1/N=N/c1cccc(C(O)=O)c1	-5.36
**4**	Nc1nc(N)nc(N)c1/N=N/c1ccccc1	-5.21
**5**	Nc1nc(N)nc(N)c1/N=N/c1cccc(C(O)=O)c1	-5.18
B096	CCC(N=C(N)N1)=C(CCc2cn(-c(cc3)ccc3[N+]([O-])=O)nn2)C1=O.Cl	-5.12
**2**	COC(c1cccc(/N=N/c2c(N)nc(N)nc2Cl)c1)=O	-5.06
**8**	CCC(N=C(N)N1)=C(Cc2cn(-c(cc3)ccc3C(O)=O)nn2)C1=O.Cl	-4.38

Figure S5: Dose-response inhibition for compound **14**.

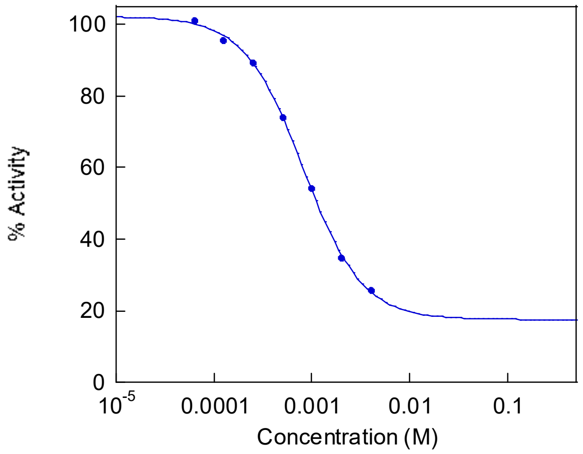

Figure S6. Superimposition of *Pf*HPPK structures in the apo form (purple) and HMDP-bound form (cyan). Region presenting high RMSD between the two forms are represented in yellow and pink, respectively. Substrates and bound Mg^{2+} ions are in green.

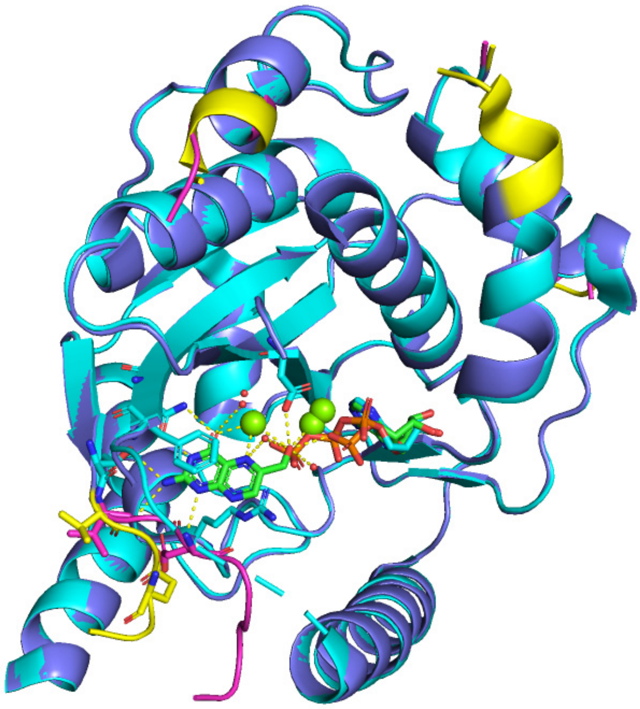

Supplement: Supplementary file 1 [file molecules-27-03515-s001.zip › molecules-1721349-supplementary.pdf]
